# 3β,6β-Diacet­oxy-5,9α-dihy­droxy-5α-cholest-7-en-11-one acetic acid 0.04-solvate

**DOI:** 10.1107/S1600536813012646

**Published:** 2013-05-11

**Authors:** Vincenzo Piccialli, Giorgia Oliviero, Nicola Borbone, Roberto Centore, Angela Tuzi

**Affiliations:** a Dipartimento di Scienze Chimiche, Università degli Studi di Napoli ’Federico II’, Complesso di Monte S. Angelo, Via Cinthia, 80126 Napoli, Italy

## Abstract

The title compound, C_31_H_48_O_7_·0.04CH_3_COOH, is a polyoxy­genated steroid obtained by selective chemical oxidation of 7-de­hydro­cholesteryl acetate. The asymmetric unit comprises three mol­ecules of the steroid (*Z*′ = 3) and a mol­ecule of acetic acid which has occupancy factor 0.131 (5). The geometric parameters of the independent mol­ecules do not reveal significant differences. In one mol­ecule, the terminal isopropyl group is disordered over two sets of sites with occupancy ratio 0.869 (5):0.131 (5). The three mol­ecules reveal different hydrogen-bonding patterns. Each of them is involved in an intra­molecular *S*(6) hydrogen-bonding motif, involving hy­droxy groups as donor and acceptor. In the crystal, two independent mol­ecules form dimers through hydrogen bonding between an OH donor and an acetate carbonyl acceptor, giving rise to *R*_2_^2^(16) ring patterns. A single hydrogen bond between the OH group and a ketone carbonyl group is observed between two symmetry-independent mol­ecules.

## Related literature

For general information on the isolation of polyoxygenated steroids from marine source, see: Piccialli & Sica (1986 ▶, 1987 ▶); Migliuolo *et al.* (1990 ▶); Notaro *et al.* (1991 ▶, 1992 ▶). For the synthesis of polyoxygenated steroids, see: Madaio *et al.* (1988 ▶); Migliuolo *et al.* (1992 ▶). For new selective oxidation protocols, see: Piccialli *et al.* (1993 ▶, 2013 ▶); Notaro *et al.* (1994 ▶); Bifulco *et al.* (2003 ▶); Caserta *et al.* (2005 ▶). For the cytotoxic activity of polyoxygenated steroids, see: Chen *et al.* (2011 ▶). For a general survey of hydrogen bonding and hydrogen-bonding synthons in crystals, see: Allen *et al.* (1999 ▶); Steiner (2002 ▶). For the graph-set analysis of hydrogen bonding, see: Bernstein *et al.* (1995 ▶). For recent examples of hydrogen bonding in crystals, see: Centore, Fusco, Jazbinsek *et al.* (2013 ▶); Centore *et al.* (2013*a* ▶,*b* ▶); Centore, Fusco, Capobianco *et al.* (2013 ▶). For the presence of multiple mol­ecules in the asymmetric unit, see: Desiraju (2007 ▶).
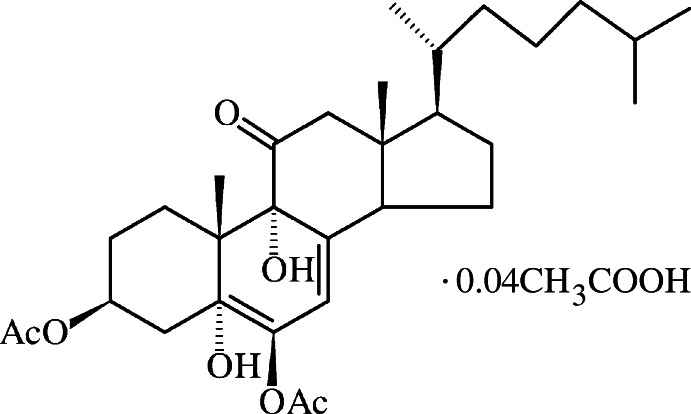


## Experimental

### 

#### Crystal data

C_31_H_48_O_7_·0.04C_2_H_4_O_2_
*M* *_r_* = 535.45
Orthorhombic, 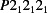

*a* = 10.242 (2) Å
*b* = 28.556 (5) Å
*c* = 30.214 (6) Å
*V* = 8837 (3) Å^3^
*Z* = 12
Mo *K*α radiation
μ = 0.08 mm^−1^
*T* = 173 K
0.45 × 0.30 × 0.17 mm

#### Data collection

Bruker–Nonius KappaCCD diffractometer
Absorption correction: multi-scan (*SADABS*; Bruker, 2001 ▶) *T*_min_ = 0.963, *T*_max_ = 0.986
82574 measured reflections
11048 independent reflections
6969 reflections with *I* > 2σ(*I*)
*R*_int_ = 0.097

#### Refinement

*R*[*F* ^2^ > 2σ(*F* ^2^)] = 0.063
*wR*(*F* ^2^) = 0.185
*S* = 1.00
11048 reflections
1072 parameters
13 restraints
H-atom parameters constrained
Δρ_max_ = 0.29 e Å^−3^
Δρ_min_ = −0.31 e Å^−3^

### 

Data collection: *COLLECT* (Nonius, 1999 ▶); cell refinement: *DIRAX/LSQ* (Duisenberg *et al.*, 2000 ▶); data reduction: *EVALCCD* (Duisenberg *et al.*, 2003 ▶); program(s) used to solve structure: *SIR97* (Altomare *et al.*, 1999 ▶); program(s) used to refine structure: *SHELXL97* (Sheldrick, 2008 ▶); molecular graphics: *ORTEP-3 for Windows* (Farrugia, 2012 ▶) and *Mercury* (Macrae *et al.*, 2006 ▶); software used to prepare material for publication: *WinGX* (Farrugia, 2012 ▶).

## Supplementary Material

Crystal structure: contains datablock(s) global, I. DOI: 10.1107/S1600536813012646/kp2452sup1.cif

Structure factors: contains datablock(s) I. DOI: 10.1107/S1600536813012646/kp2452Isup2.hkl

Additional supplementary materials:  crystallographic information; 3D view; checkCIF report

## Figures and Tables

**Table 1:** Hydrogen-bond geometry (Å, °)

*D*—H⋯*A*	*D*—H	H⋯*A*	*D*⋯*A*	*D*—H⋯*A*
O1*A*—H1*AO*⋯O3*C* ^i^	0.95	1.79	2.745 (4)	177
O1*B*—H1*BO*⋯O5*C*	0.80	2.02	2.819 (4)	175
O1*C*—H1*CO*⋯O5*B*	0.89	1.81	2.699 (4)	178
O2*A*—H2*AO*⋯O1*A*	0.93	1.88	2.691 (4)	145
O2*B*—H2*BO*⋯O1*B*	0.90	1.80	2.628 (4)	153
O2*C*—H2*CO*⋯O1*C*	1.05	1.77	2.614 (4)	134

Symmetry code: (i) 
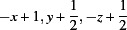
.

**Fig. 1.**
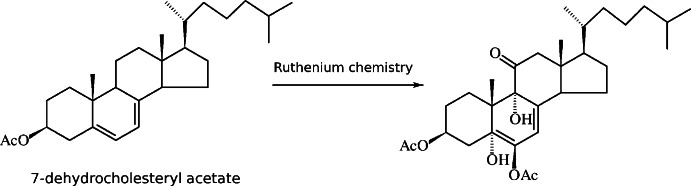
Synthesis of the title compound by ruthenium catalyzed selective oxidation of 7-dehydrocholesteryl acetate.

**Fig. 2.**
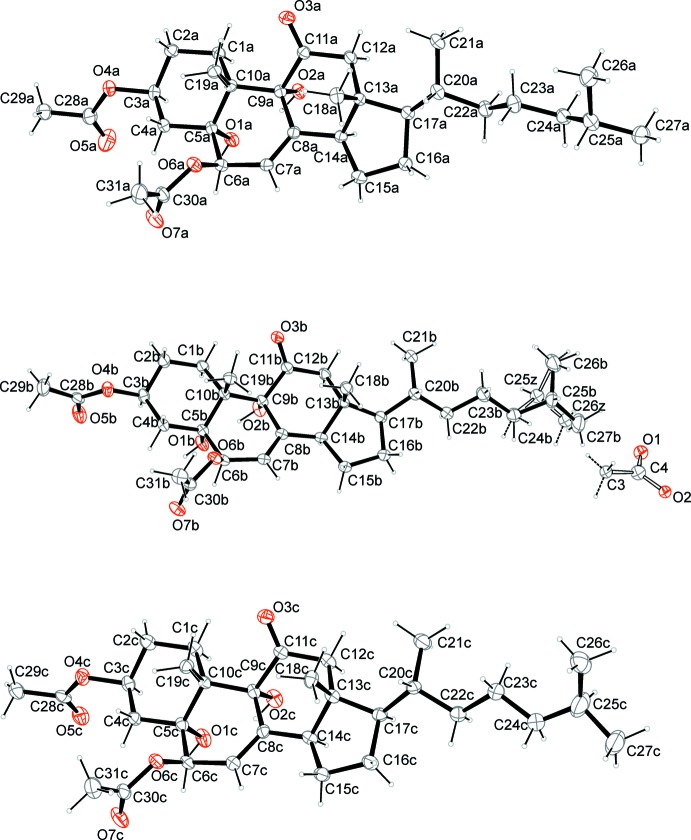
ORTEP views of the three independent molecules, thermal ellipsoids are drawn at 30% probability level. In the B molecule, the terminal isopropyl group refined with the minor occupancy factor and the solvent molecule are drawn in open line.

**Fig. 3.**
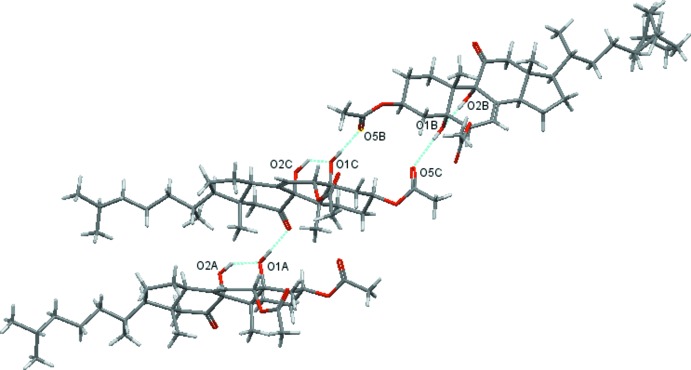
H-bonding pattern joining the three independent molecules. The H-bonds are shown as dashed lines.

## supplementary crystallographic information

### Comment

Polyoxygenated steroids possessing a wide range of oxygenation and nuclear substitution patterns have been isolated from a variety of marine organisms. Some of them show antitumour and antinflammatory activities as well as other biological effects. Thus, the research in this field is still very active. Previous studies in our group focused on the isolation of polyoxygenated steroids from marine sources (Piccialli & Sica, 1986, 1987; Notaro *et al.*, 1991) as well as on the synthesis (Madaio, Piccialli & Sica, 1988; Migliuolo *et al.*, 1992) of some of them exploiting new ruthenium tetroxide oxygenation protocols developed in our laboratories (Piccialli *et al.*, 2013; Notaro *et al.*, 1994). As a continuation of our interest in this field, we have now undertaken a study aimed at preparing a new collection of polyoxygenated steroids for structure-activity relationship studies and in particular at the synthesis of new 9,11-secosteroids. Many representatives of this sub-class of steroids have been shown to possess *in vitro* cytotoxic activity against various human cancer cell lines (Chen *et al.*, 2011). Due to its functionalization pattern, the title compound, shown in the Scheme, is a good starting product to further oxygenate the steroid nucleus at C and D rings, as well as to obtain the 9,11-secosteroid motif. For now, the title compound has been synthesized starting from commercially available 7-dehydrocholesteryl acetate (Fig. 1).

The X-ray analysis of the title compound confirms the structure and stereochemistry of the synthesized compound. The asymmetric unit contains three independent molecules that have similar geometric parameters (Fig. 2). Rings A and C of the steroid skeleton adopt a chair conformation. Ring B, which contains a double bond at C7=C8, adopts a half- chair conformation with twist at C5—C10 bond. The five-membered D ring is in envelope conformation, with C13 at the flap. In the steroid core *trans* junctions at A/B and C/D rings are observed. The acetyl group at C3 (ring A) is equatorial while acetyl group at C6 (ring B) is axial. The lateral alkyl chain is fully extended. In one molecule, two different positions were found for the isopropyl group of the lateral alkylic chain (occupancy factor refined to 0.869 (5) for C25B/C26B/C27B; 0.131 (5) for C25*Z*/C26*Z*/C27*Z*). In the crystal, acetic acid solvent of crystallization was found with occupancy factor refined to 0.131 (5).

The molecule of the title compound contains several H–bonding donor and acceptor groups (Allen *et al.*, 1999; Steiner, 2002) that are responsible for the formation of H bonds (Centore *et al.*, 2013*a*, 2013*b*; Centore, Fusco, Jazbinsek *et al.* 2013; Centore, Fusco, Capobianco *et al.* 2013). The three independent molecules A, B and C are involved in intra and intermolecular H bonding patterns (Fig. 3). In each of the three independent molecules there is an intramolecular H bonding motif S(6) between hydroxy O2 donor and hydroxy O1 acceptor of the same molecule. Molecules B and C create dimers through intermolecular H bonding between OH donors (O1B and O1C) and acetate carbonyl acceptors (O5C and O5B), giving rise to ring patterns *R*^2^_2_(16). Molecules C and A are involved in a single intermolecular H bond between OH donor (O1A) and ketone carbonyl acceptor (O3C).

### Experimental

The title compound was synthesized starting from commercially available 7-dehydrocholesteryl acetate according to a procedure already described (Notaro *et al.*, 1994). Crystals suitable for X-ray analysis were obtained from acetic acid solution by slow evaporation of the solvent.

### Refinement

The H atom of the hydroxy groups were located in difference map with exception of carboxy H atom of the acetic acid solvent molecule that was not assigned. Other H atoms were generated stereochemically. All H atoms were refined by the riding model with *U*_iso_=1.2×*U*_eq_ of the carrier atom (1.5 for H atoms of the methyl groups). Some reflections with theta below 3° were removed from the final refinement (*OMIT* instruction) because their intensities were strongly affected by the beamstop. Some constraints were introduced in the refinement to handle the disorder of the terminal isopropyl group of molecule B. In the absence of strong anomalous scatterer the Flack parameter is not meaningful. Data were merged using MERG 3 instruction and the absolute configuration was assigned on the basis of the known chirality of the precursor used in the synthesis.

### Crystal data

**Table:**

C_31_H_48_O_7_·0.04C_2_H_4_O_2_	*F*(000) = 3498
*M**_r_* = 535.45	*D*_x_ = 1.207 Mg m^−^^3^
Orthorhombic, *P*2_1_2_1_2_1_	Mo *K*α radiation, λ = 0.71073 Å
Hall symbol: P 2ac 2ab	Cell parameters from 454 reflections
*a* = 10.242 (2) Å	θ = 4.9–20.8°
*b* = 28.556 (5) Å	µ = 0.08 mm^−^^1^
*c* = 30.214 (6) Å	*T* = 173 K
*V* = 8837 (3) Å^3^	Block, colorless
*Z* = 12	0.45 × 0.30 × 0.17 mm

### Data collection

**Table:**

Bruker–Nonius KappaCCD diffractometer	11048 independent reflections
Radiation source: normal-focus sealed tube	6969 reflections with *I* > 2σ(*I*)
Graphite monochromator	*R*_int_ = 0.097
Detector resolution: 9 pixels mm^-1^	θ_max_ = 27.5°, θ_min_ = 2.1°
CCD rotation images, thick slices scans	*h* = −13→13
Absorption correction: multi-scan (*SADABS*; Bruker, 2001)	*k* = −35→37
*T*_min_ = 0.963, *T*_max_ = 0.986	*l* = −39→38
82574 measured reflections	

### Refinement

**Table:**

Refinement on *F*^2^	Primary atom site location: structure-invariant direct methods
Least-squares matrix: full	Secondary atom site location: difference Fourier map
*R*[*F*^2^ > 2σ(*F*^2^)] = 0.063	Hydrogen site location: inferred from neighbouring sites
*wR*(*F*^2^) = 0.185	H-atom parameters constrained
*S* = 1.00	*w* = 1/[σ^2^(*F*_o_^2^) + (0.1168*P*)^2^] where *P* = (*F*_o_^2^ + 2*F*_c_^2^)/3
11048 reflections	(Δ/σ)_max_ = 0.004
1072 parameters	Δρ_max_ = 0.29 e Å^−^^3^
13 restraints	Δρ_min_ = −0.31 e Å^−^^3^

### Special details

**Table:**

Geometry. All e.s.d.'s (except the e.s.d. in the dihedral angle between two l.s. planes) are estimated using the full covariance matrix. The cell e.s.d.'s are taken into account individually in the estimation of e.s.d.'s in distances, angles and torsion angles; correlations between e.s.d.'s in cell parameters are only used when they are defined by crystal symmetry. An approximate (isotropic) treatment of cell e.s.d.'s is used for estimating e.s.d.'s involving l.s. planes.
Refinement. Refinement of *F*^2^ against ALL reflections. The weighted *R*-factor *wR* and goodness of fit *S* are based on *F*^2^, conventional *R*-factors *R* are based on *F*, with *F* set to zero for negative *F*^2^. The threshold expression of *F*^2^ > σ(*F*^2^) is used only for calculating *R*-factors(gt) *etc*. and is not relevant to the choice of reflections for refinement. *R*-factors based on *F*^2^ are statistically about twice as large as those based on *F*, and *R*- factors based on ALL data will be even larger.

### Fractional atomic coordinates and isotropic or equivalent isotropic displacement parameters (Å^2^)

**Table:**

	*x*	*y*	*z*	*U*_iso_*/*U*_eq_	Occ. (<1)
C1A	0.9865 (4)	0.37429 (15)	0.18977 (14)	0.0322 (10)	
H1A1	1.0372	0.3464	0.1988	0.039*	
H1A2	1.0378	0.3913	0.1671	0.039*	
C1B	0.2980 (5)	0.42430 (15)	0.42610 (15)	0.0399 (11)	
H1B1	0.2578	0.4507	0.4423	0.048*	
H1B2	0.2267	0.4051	0.4134	0.048*	
C1C	0.2794 (4)	0.11749 (15)	0.36525 (15)	0.0387 (11)	
H1C1	0.2328	0.0900	0.3531	0.046*	
H1C2	0.2144	0.1373	0.3807	0.046*	
C2A	0.9669 (4)	0.40597 (15)	0.22994 (14)	0.0350 (10)	
H2A1	0.9209	0.3884	0.2535	0.042*	
H2A2	1.0530	0.4158	0.2416	0.042*	
C2B	0.3766 (5)	0.39443 (16)	0.45845 (17)	0.0464 (12)	
H2B1	0.4440	0.4140	0.4729	0.056*	
H2B2	0.3180	0.3821	0.4818	0.056*	
C2C	0.3383 (5)	0.14542 (15)	0.32700 (16)	0.0412 (11)	
H2C1	0.3995	0.1254	0.3101	0.049*	
H2C2	0.2682	0.1558	0.3067	0.049*	
C3A	0.8882 (5)	0.44887 (14)	0.21755 (14)	0.0339 (10)	
H3A	0.9384	0.4685	0.1961	0.041*	
C3B	0.4408 (4)	0.35442 (15)	0.43489 (16)	0.0381 (11)	
H3B	0.3728	0.3323	0.4236	0.046*	
C3C	0.4097 (5)	0.18743 (15)	0.34513 (15)	0.0378 (11)	
H3C	0.3463	0.2097	0.3589	0.045*	
C4A	0.7591 (4)	0.43526 (14)	0.19780 (14)	0.0313 (9)	
H4A1	0.7122	0.4638	0.1882	0.038*	
H4A2	0.7054	0.4196	0.2206	0.038*	
C4B	0.5264 (4)	0.37100 (14)	0.39673 (14)	0.0331 (10)	
H4B1	0.5617	0.3434	0.3809	0.040*	
H4B2	0.6011	0.3890	0.4087	0.040*	
C4C	0.5132 (4)	0.17375 (14)	0.37843 (14)	0.0347 (10)	
H4C1	0.5532	0.2024	0.3909	0.042*	
H4C2	0.5827	0.1558	0.3632	0.042*	
C5A	0.7760 (4)	0.40225 (14)	0.15796 (13)	0.0304 (9)	
C5B	0.4507 (4)	0.40168 (14)	0.36422 (14)	0.0306 (9)	
C5C	0.4572 (4)	0.14427 (14)	0.41599 (15)	0.0332 (10)	
C6A	0.6421 (4)	0.39143 (14)	0.13707 (14)	0.0333 (10)	
H6A	0.6097	0.4204	0.1221	0.040*	
C6B	0.5359 (4)	0.41803 (15)	0.32564 (14)	0.0313 (10)	
H6B	0.5444	0.3912	0.3045	0.038*	
C6C	0.5675 (5)	0.13146 (16)	0.44961 (15)	0.0414 (12)	
H6C	0.5861	0.1600	0.4678	0.050*	
C7A	0.6504 (4)	0.35362 (14)	0.10349 (14)	0.0325 (10)	
H7A	0.5759	0.3480	0.0856	0.039*	
C7B	0.4826 (4)	0.45851 (15)	0.30054 (14)	0.0349 (10)	
H7B	0.5292	0.4685	0.2750	0.042*	
C7C	0.5268 (5)	0.09265 (16)	0.48091 (15)	0.0400 (11)	
H7C	0.5836	0.0851	0.5047	0.048*	
C8A	0.7566 (4)	0.32687 (13)	0.09680 (13)	0.0292 (9)	
C8B	0.3742 (4)	0.48185 (14)	0.31118 (14)	0.0308 (9)	
C8C	0.4142 (4)	0.06810 (14)	0.47696 (13)	0.0313 (10)	
C9A	0.8781 (4)	0.32994 (13)	0.12531 (13)	0.0279 (9)	
C9B	0.2987 (4)	0.47081 (14)	0.35379 (14)	0.0318 (10)	
C9C	0.3169 (4)	0.07645 (13)	0.43965 (14)	0.0313 (10)	
C10A	0.8560 (4)	0.35833 (13)	0.16923 (13)	0.0271 (9)	
C10B	0.3832 (4)	0.44372 (14)	0.38819 (14)	0.0321 (10)	
C10C	0.3815 (4)	0.10057 (13)	0.39901 (14)	0.0291 (9)	
C11A	0.9371 (4)	0.28079 (14)	0.13319 (14)	0.0302 (9)	
C11B	0.2357 (4)	0.51585 (14)	0.37272 (15)	0.0372 (11)	
C11C	0.2459 (4)	0.02994 (14)	0.42841 (14)	0.0335 (10)	
C12A	0.9345 (5)	0.24626 (14)	0.09543 (14)	0.0337 (10)	
H12A	0.9578	0.2148	0.1068	0.040*	
H12B	1.0012	0.2554	0.0733	0.040*	
C12B	0.1645 (5)	0.54625 (15)	0.33994 (15)	0.0398 (11)	
H12C	0.1281	0.5741	0.3551	0.048*	
H12D	0.0913	0.5285	0.3266	0.048*	
C12C	0.1979 (5)	0.00168 (15)	0.46683 (15)	0.0377 (11)	
H12E	0.1541	−0.0270	0.4561	0.045*	
H12F	0.1339	0.0201	0.4841	0.045*	
C13A	0.8006 (4)	0.24362 (13)	0.07263 (13)	0.0301 (9)	
C13B	0.2604 (4)	0.56181 (14)	0.30351 (14)	0.0316 (9)	
C13C	0.3142 (4)	−0.01141 (14)	0.49610 (14)	0.0312 (10)	
C14A	0.7657 (4)	0.29391 (13)	0.05852 (13)	0.0303 (9)	
H14A	0.8397	0.3052	0.0398	0.036*	
C14B	0.3091 (4)	0.51648 (15)	0.28100 (13)	0.0337 (10)	
H14B	0.2292	0.5004	0.2695	0.040*	
C14C	0.3758 (5)	0.03435 (14)	0.51265 (14)	0.0346 (10)	
H14C	0.3049	0.0505	0.5295	0.041*	
C15A	0.6501 (5)	0.28780 (15)	0.02776 (15)	0.0413 (11)	
H15A	0.6434	0.3144	0.0068	0.050*	
H15B	0.5676	0.2854	0.0447	0.050*	
C15B	0.3821 (5)	0.53441 (16)	0.24014 (15)	0.0462 (12)	
H15E	0.3842	0.5104	0.2165	0.055*	
H15F	0.4727	0.5434	0.2477	0.055*	
C15C	0.4737 (5)	0.01892 (15)	0.54743 (14)	0.0379 (10)	
H15C	0.4911	0.0443	0.5689	0.046*	
H15D	0.5571	0.0091	0.5337	0.046*	
C16A	0.6796 (5)	0.24186 (15)	0.00312 (15)	0.0400 (11)	
H16A	0.7032	0.2484	−0.0280	0.048*	
H16B	0.6020	0.2212	0.0034	0.048*	
C16B	0.3010 (5)	0.57762 (17)	0.22566 (15)	0.0456 (12)	
H16C	0.3587	0.6051	0.2214	0.055*	
H16D	0.2553	0.5711	0.1975	0.055*	
C16C	0.4055 (5)	−0.02247 (16)	0.56966 (15)	0.0399 (11)	
H16E	0.3782	−0.0139	0.6000	0.048*	
H16F	0.4654	−0.0496	0.5714	0.048*	
C17A	0.7960 (4)	0.21796 (14)	0.02762 (13)	0.0316 (10)	
H17A	0.8774	0.2264	0.0112	0.038*	
C17B	0.2012 (5)	0.58737 (15)	0.26287 (14)	0.0365 (10)	
H17B	0.1193	0.5703	0.2548	0.044*	
C17C	0.2833 (4)	−0.03506 (14)	0.54091 (13)	0.0322 (10)	
H17C	0.2075	−0.0178	0.5539	0.039*	
C18A	0.7001 (5)	0.22420 (14)	0.10532 (14)	0.0358 (10)	
H18A	0.7248	0.1923	0.1139	0.054*	
H18B	0.6138	0.2237	0.0913	0.054*	
H18C	0.6972	0.2442	0.1317	0.054*	
C18B	0.3712 (5)	0.58937 (16)	0.32442 (16)	0.0415 (11)	
H18D	0.4292	0.6012	0.3012	0.062*	
H18E	0.4206	0.5689	0.3444	0.062*	
H18F	0.3353	0.6158	0.3412	0.062*	
C18C	0.4115 (5)	−0.04103 (15)	0.47005 (15)	0.0371 (11)	
H18G	0.4505	−0.0220	0.4465	0.056*	
H18H	0.3661	−0.0679	0.4569	0.056*	
H18I	0.4802	−0.0522	0.4900	0.056*	
C19A	0.7801 (4)	0.32707 (14)	0.20199 (13)	0.0320 (10)	
H19G	0.8397	0.3041	0.2151	0.048*	
H19H	0.7100	0.3107	0.1862	0.048*	
H19I	0.7424	0.3466	0.2254	0.048*	
C19B	0.4890 (4)	0.47704 (15)	0.40673 (15)	0.0362 (10)	
H19A	0.5268	0.4953	0.3825	0.054*	
H19B	0.5578	0.4586	0.4211	0.054*	
H19C	0.4495	0.4983	0.4284	0.054*	
C19C	0.4764 (4)	0.06638 (14)	0.37703 (14)	0.0330 (10)	
H19D	0.5222	0.0484	0.3999	0.050*	
H19E	0.5401	0.0839	0.3594	0.050*	
H19F	0.4278	0.0450	0.3578	0.050*	
C20A	0.7851 (5)	0.16422 (14)	0.02699 (14)	0.0345 (10)	
H20A	0.6988	0.1557	0.0402	0.041*	
C20B	0.1652 (5)	0.63964 (15)	0.26693 (15)	0.0405 (11)	
H20B	0.2476	0.6575	0.2725	0.049*	
C20C	0.2483 (5)	−0.08717 (14)	0.54216 (13)	0.0338 (10)	
H20C	0.3233	−0.1049	0.5293	0.041*	
C21A	0.8898 (5)	0.14014 (15)	0.05398 (16)	0.0425 (12)	
H21D	0.8852	0.1063	0.0491	0.064*	
H21E	0.8762	0.1468	0.0855	0.064*	
H21F	0.9758	0.1517	0.0449	0.064*	
C21B	0.0726 (6)	0.64996 (17)	0.30456 (17)	0.0556 (14)	
H21G	−0.0058	0.6305	0.3015	0.083*	
H21H	0.0479	0.6831	0.3038	0.083*	
H21I	0.1155	0.6429	0.3328	0.083*	
C21C	0.1283 (5)	−0.09820 (17)	0.51466 (17)	0.0514 (13)	
H21A	0.0565	−0.0776	0.5235	0.077*	
H21B	0.1028	−0.1309	0.5194	0.077*	
H21C	0.1480	−0.0933	0.4833	0.077*	
C22A	0.7871 (5)	0.14571 (15)	−0.02089 (14)	0.0398 (11)	
H22A	0.7386	0.1678	−0.0401	0.048*	
H22B	0.8785	0.1446	−0.0315	0.048*	
C22B	0.1075 (5)	0.65710 (16)	0.22319 (15)	0.0452 (12)	
H22C	0.1710	0.6504	0.1993	0.054*	
H22D	0.0277	0.6388	0.2168	0.054*	
C22C	0.2307 (5)	−0.10359 (16)	0.59008 (15)	0.0448 (12)	
H22E	0.3114	−0.0960	0.6066	0.054*	
H22F	0.1589	−0.0852	0.6035	0.054*	
C23A	0.7274 (5)	0.09747 (15)	−0.02517 (15)	0.0400 (11)	
H23A	0.6339	0.0995	−0.0171	0.048*	
H23B	0.7702	0.0764	−0.0035	0.048*	
C23B	0.0727 (6)	0.70907 (17)	0.22135 (18)	0.0574 (14)	
H23C	0.0047	0.7155	0.2438	0.069*	
H23D	0.1510	0.7276	0.2294	0.069*	
C23C	0.2013 (6)	−0.15500 (17)	0.59671 (15)	0.0489 (13)	
H23E	0.1117	−0.1613	0.5859	0.059*	
H23F	0.2621	−0.1734	0.5781	0.059*	
C24A	0.7379 (5)	0.07539 (15)	−0.07094 (15)	0.0390 (11)	
H24A	0.8298	0.0661	−0.0761	0.047*	
H24B	0.7149	0.0992	−0.0934	0.047*	
C24C	0.2115 (6)	−0.17211 (19)	0.64309 (16)	0.0576 (14)	
H24C	0.3048	−0.1700	0.6514	0.069*	
H24D	0.1644	−0.1492	0.6618	0.069*	
C25A	0.6507 (5)	0.03259 (15)	−0.07753 (16)	0.0434 (12)	
H25A	0.5581	0.0430	−0.0739	0.052*	
C25C	0.1671 (10)	−0.2182 (2)	0.65672 (18)	0.089 (2)	
H25C	0.0708	−0.2128	0.6589	0.107*	
C26A	0.6777 (6)	−0.00509 (17)	−0.04207 (17)	0.0555 (15)	
H26A	0.6203	−0.0320	−0.0470	0.083*	
H26B	0.6611	0.0080	−0.0126	0.083*	
H26C	0.7690	−0.0151	−0.0441	0.083*	
C26C	0.1710 (7)	−0.25678 (18)	0.6255 (2)	0.0674 (17)	
H26D	0.1382	−0.2462	0.5967	0.101*	
H26E	0.1164	−0.2825	0.6364	0.101*	
H26F	0.2612	−0.2677	0.6223	0.101*	
C27A	0.6643 (5)	0.01231 (17)	−0.12347 (16)	0.0507 (13)	
H27A	0.6442	0.0365	−0.1455	0.076*	
H27B	0.6036	−0.0140	−0.1269	0.076*	
H27C	0.7540	0.0012	−0.1278	0.076*	
C27C	0.1988 (7)	−0.2302 (2)	0.70375 (18)	0.0783 (19)	
H27G	0.1487	−0.2578	0.7128	0.117*	
H27H	0.1762	−0.2037	0.7229	0.117*	
H27I	0.2924	−0.2368	0.7064	0.117*	
C28A	0.8326 (5)	0.52074 (17)	0.25385 (17)	0.0476 (13)	
C28B	0.5104 (5)	0.28518 (15)	0.47368 (15)	0.0376 (11)	
C28C	0.4840 (5)	0.25682 (15)	0.30774 (16)	0.0382 (11)	
C29A	0.7923 (6)	0.54130 (17)	0.29682 (17)	0.0516 (13)	
H29A	0.7662	0.5740	0.2924	0.077*	
H29B	0.8656	0.5400	0.3176	0.077*	
H29C	0.7185	0.5235	0.3088	0.077*	
C29B	0.5981 (6)	0.26760 (17)	0.50848 (18)	0.0584 (15)	
H29D	0.6506	0.2935	0.5201	0.088*	
H29E	0.6560	0.2437	0.4960	0.088*	
H29F	0.5463	0.2539	0.5325	0.088*	
C29C	0.5692 (6)	0.27443 (18)	0.27093 (18)	0.0546 (14)	
H29G	0.5257	0.3006	0.2560	0.082*	
H29H	0.5846	0.2491	0.2497	0.082*	
H29I	0.6527	0.2851	0.2831	0.082*	
C30A	0.4499 (5)	0.40755 (19)	0.17975 (17)	0.0444 (12)	
C30B	0.7633 (4)	0.39888 (16)	0.33229 (15)	0.0356 (10)	
C30C	0.7866 (5)	0.14783 (18)	0.43033 (16)	0.0459 (13)	
C31A	0.3517 (5)	0.3866 (2)	0.20930 (19)	0.0591 (15)	
H31G	0.3120	0.4111	0.2275	0.089*	
H31H	0.3937	0.3634	0.2285	0.089*	
H31I	0.2839	0.3713	0.1915	0.089*	
C31B	0.8882 (5)	0.41365 (19)	0.35334 (18)	0.0481 (13)	
H31A	0.8761	0.4440	0.3679	0.072*	
H31B	0.9562	0.4163	0.3307	0.072*	
H31C	0.9145	0.3903	0.3754	0.072*	
C31C	0.9060 (5)	0.12716 (19)	0.40886 (19)	0.0561 (14)	
H31D	0.9778	0.1498	0.4102	0.084*	
H31E	0.8867	0.1197	0.3779	0.084*	
H31F	0.9312	0.0985	0.4245	0.084*	
O1A	0.8478 (3)	0.42643 (9)	0.12391 (9)	0.0362 (7)	
H1AO	0.8232	0.4582	0.1187	0.043*	
O1B	0.3478 (3)	0.37467 (10)	0.34475 (11)	0.0405 (8)	
H1BO	0.3667	0.3476	0.3413	0.049*	
O1C	0.3636 (3)	0.17171 (10)	0.43997 (11)	0.0485 (9)	
H1CO	0.3892	0.2013	0.4438	0.058*	
O2A	0.9809 (3)	0.34985 (10)	0.09829 (9)	0.0361 (7)	
H2AO	0.9555	0.3810	0.0977	0.043*	
O2B	0.1830 (3)	0.44513 (11)	0.34136 (12)	0.0452 (8)	
H2BO	0.2168	0.4162	0.3403	0.054*	
O2C	0.2089 (3)	0.10155 (11)	0.45839 (11)	0.0462 (8)	
H2CO	0.2334	0.1372	0.4608	0.055*	
O3A	0.9891 (3)	0.27009 (10)	0.16771 (11)	0.0440 (8)	
O3B	0.2412 (4)	0.52654 (11)	0.41166 (11)	0.0510 (9)	
O3C	0.2310 (3)	0.01689 (11)	0.39123 (10)	0.0456 (8)	
O4A	0.8634 (3)	0.47536 (10)	0.25800 (9)	0.0385 (7)	
O4B	0.5250 (3)	0.33030 (10)	0.46650 (10)	0.0407 (8)	
O4C	0.4774 (3)	0.21033 (10)	0.30791 (10)	0.0427 (8)	
O5A	0.8372 (6)	0.54073 (13)	0.21993 (13)	0.0853 (16)	
O5B	0.4347 (4)	0.26170 (11)	0.45286 (13)	0.0602 (10)	
O5C	0.4292 (4)	0.28112 (11)	0.33343 (13)	0.0537 (9)	
O6A	0.5444 (3)	0.37677 (11)	0.16954 (10)	0.0388 (7)	
O6B	0.6680 (3)	0.43061 (10)	0.34042 (10)	0.0352 (7)	
O6C	0.6855 (3)	0.11783 (10)	0.42892 (10)	0.0400 (8)	
O7A	0.4481 (4)	0.44727 (14)	0.16561 (14)	0.0640 (11)	
O7B	0.7464 (3)	0.36348 (11)	0.31171 (11)	0.0467 (8)	
O7C	0.7797 (4)	0.18617 (13)	0.44649 (14)	0.0630 (11)	
C24B	0.0242 (7)	0.72549 (18)	0.17701 (16)	0.0579 (15)	0.869 (5)
H24E	−0.0579	0.7085	0.1705	0.069*	0.869 (5)
H24F	0.0889	0.7159	0.1544	0.069*	0.869 (5)
C25B	−0.0018 (7)	0.77782 (19)	0.17113 (19)	0.0556 (17)	0.869 (5)
H25B	0.0825	0.7949	0.1752	0.067*	0.869 (5)
C26B	−0.0958 (7)	0.7950 (2)	0.2048 (2)	0.079 (2)	0.869 (5)
H26G	−0.1791	0.7785	0.2014	0.118*	0.869 (5)
H26H	−0.1097	0.8287	0.2008	0.118*	0.869 (5)
H26I	−0.0607	0.7892	0.2345	0.118*	0.869 (5)
C27B	−0.0514 (10)	0.7879 (2)	0.1256 (2)	0.085 (3)	0.869 (5)
H27D	−0.1186	0.7650	0.1177	0.128*	0.869 (5)
H27E	0.0209	0.7860	0.1044	0.128*	0.869 (5)
H27F	−0.0890	0.8195	0.1248	0.128*	0.869 (5)
C24Z	0.0242 (7)	0.72549 (18)	0.17701 (16)	0.0579 (15)	0.131 (5)
H24G	0.0139	0.6977	0.1578	0.069*	0.131 (5)
H24H	0.0928	0.7454	0.1636	0.069*	0.131 (5)
C25Z	−0.1036 (17)	0.7528 (6)	0.1760 (6)	0.049 (10)*	0.131 (5)
H25Z	−0.1756	0.7322	0.1869	0.059*	0.131 (5)
C26Z	−0.132 (3)	0.7669 (11)	0.1295 (6)	0.047 (10)*	0.131 (5)
H26J	−0.2262	0.7725	0.1263	0.070*	0.131 (5)
H26K	−0.1054	0.7418	0.1094	0.070*	0.131 (5)
H26L	−0.0843	0.7956	0.1224	0.070*	0.131 (5)
C27Z	−0.0958 (7)	0.7950 (2)	0.2048 (2)	0.079 (2)	0.131 (5)
H27J	−0.0245	0.8153	0.1947	0.118*	0.131 (5)
H27K	−0.0794	0.7853	0.2354	0.118*	0.131 (5)
H27L	−0.1785	0.8123	0.2034	0.118*	0.131 (5)
O1	−0.060 (3)	0.8409 (9)	0.0564 (9)	0.049 (7)*	0.131 (5)
O2	0.116 (2)	0.8592 (7)	0.0144 (7)	0.036 (6)*	0.131 (5)
C3	0.161 (3)	0.8090 (12)	0.0734 (11)	0.038 (8)*	0.131 (5)
H3D	0.2012	0.7846	0.0550	0.057*	0.131 (5)
H3E	0.1139	0.7943	0.0979	0.057*	0.131 (5)
H3F	0.2299	0.8294	0.0853	0.057*	0.131 (5)
C4	0.071 (4)	0.8368 (14)	0.0467 (14)	0.053 (11)*	0.131 (5)

### Atomic displacement parameters (Å^2^)

**Table:**

	*U*^11^	*U*^22^	*U*^33^	*U*^12^	*U*^13^	*U*^23^
C1A	0.032 (2)	0.031 (2)	0.034 (2)	0.0028 (18)	0.002 (2)	0.0026 (19)
C1B	0.038 (3)	0.034 (2)	0.048 (3)	0.003 (2)	0.018 (2)	0.006 (2)
C1C	0.031 (3)	0.032 (2)	0.053 (3)	−0.002 (2)	−0.004 (2)	0.006 (2)
C2A	0.030 (2)	0.036 (2)	0.039 (2)	−0.0011 (19)	−0.006 (2)	−0.002 (2)
C2B	0.047 (3)	0.040 (3)	0.052 (3)	0.003 (2)	0.016 (3)	0.010 (2)
C2C	0.041 (3)	0.030 (2)	0.053 (3)	−0.002 (2)	−0.011 (2)	0.008 (2)
C3A	0.040 (3)	0.031 (2)	0.031 (2)	−0.003 (2)	0.005 (2)	−0.0055 (18)
C3B	0.033 (3)	0.030 (2)	0.052 (3)	−0.002 (2)	−0.003 (2)	0.004 (2)
C3C	0.038 (3)	0.031 (2)	0.045 (3)	−0.002 (2)	0.004 (2)	0.005 (2)
C4A	0.032 (2)	0.028 (2)	0.034 (2)	0.0011 (18)	0.002 (2)	−0.0032 (17)
C4B	0.030 (2)	0.028 (2)	0.042 (2)	0.0012 (18)	−0.002 (2)	−0.0017 (19)
C4C	0.036 (3)	0.031 (2)	0.037 (2)	−0.008 (2)	0.003 (2)	−0.0032 (19)
C5A	0.035 (2)	0.027 (2)	0.030 (2)	0.0014 (18)	0.0055 (19)	−0.0003 (17)
C5B	0.026 (2)	0.027 (2)	0.038 (2)	−0.0020 (18)	−0.0056 (19)	−0.0039 (18)
C5C	0.035 (3)	0.026 (2)	0.039 (2)	−0.0036 (19)	0.005 (2)	−0.0041 (19)
C6A	0.034 (2)	0.032 (2)	0.034 (2)	0.0065 (19)	−0.001 (2)	0.0017 (18)
C6B	0.021 (2)	0.037 (2)	0.036 (2)	0.0015 (18)	−0.0035 (19)	−0.0116 (19)
C6C	0.045 (3)	0.037 (2)	0.042 (3)	−0.019 (2)	−0.005 (2)	0.002 (2)
C7A	0.036 (3)	0.031 (2)	0.031 (2)	0.005 (2)	−0.006 (2)	0.0008 (18)
C7B	0.036 (3)	0.036 (2)	0.033 (2)	0.001 (2)	0.004 (2)	−0.0061 (18)
C7C	0.041 (3)	0.044 (3)	0.035 (2)	−0.011 (2)	−0.003 (2)	0.003 (2)
C8A	0.037 (3)	0.0239 (19)	0.027 (2)	0.0020 (19)	−0.0007 (19)	0.0034 (16)
C8B	0.027 (2)	0.031 (2)	0.035 (2)	−0.0024 (18)	0.0023 (19)	−0.0058 (19)
C8C	0.037 (3)	0.029 (2)	0.028 (2)	−0.0016 (19)	0.0096 (19)	−0.0067 (17)
C9A	0.031 (2)	0.0242 (19)	0.029 (2)	−0.0007 (18)	0.0031 (19)	0.0021 (17)
C9B	0.023 (2)	0.028 (2)	0.044 (2)	−0.0002 (18)	0.0035 (19)	−0.0003 (19)
C9C	0.031 (2)	0.0213 (19)	0.041 (2)	0.0002 (18)	0.010 (2)	−0.0071 (18)
C10A	0.028 (2)	0.028 (2)	0.026 (2)	−0.0004 (18)	0.0012 (18)	0.0025 (16)
C10B	0.028 (2)	0.027 (2)	0.041 (2)	0.0004 (18)	0.006 (2)	0.0014 (18)
C10C	0.029 (2)	0.0231 (19)	0.036 (2)	−0.0022 (18)	−0.0007 (19)	−0.0012 (17)
C11A	0.029 (2)	0.030 (2)	0.032 (2)	0.0006 (18)	0.003 (2)	0.0013 (18)
C11B	0.033 (3)	0.032 (2)	0.046 (3)	0.001 (2)	0.018 (2)	0.004 (2)
C11C	0.032 (2)	0.030 (2)	0.039 (2)	−0.0027 (19)	−0.002 (2)	−0.0033 (19)
C12A	0.037 (3)	0.027 (2)	0.037 (2)	0.0064 (19)	0.002 (2)	−0.0011 (18)
C12B	0.036 (3)	0.035 (2)	0.048 (3)	0.008 (2)	0.012 (2)	0.003 (2)
C12C	0.036 (3)	0.028 (2)	0.049 (3)	−0.0061 (19)	−0.001 (2)	−0.004 (2)
C13A	0.033 (2)	0.027 (2)	0.030 (2)	0.0062 (18)	0.0003 (19)	0.0000 (17)
C13B	0.031 (2)	0.030 (2)	0.034 (2)	0.0049 (18)	0.004 (2)	−0.0036 (18)
C13C	0.025 (2)	0.027 (2)	0.041 (2)	0.0017 (18)	0.000 (2)	−0.0052 (18)
C14A	0.036 (2)	0.026 (2)	0.028 (2)	0.0029 (19)	−0.0025 (19)	0.0014 (17)
C14B	0.033 (2)	0.036 (2)	0.032 (2)	0.001 (2)	0.002 (2)	−0.0091 (19)
C14C	0.032 (2)	0.029 (2)	0.043 (2)	−0.0027 (19)	−0.004 (2)	−0.0051 (19)
C15A	0.049 (3)	0.033 (2)	0.042 (3)	0.011 (2)	−0.006 (2)	0.003 (2)
C15B	0.056 (3)	0.046 (3)	0.036 (3)	0.017 (2)	0.009 (2)	−0.002 (2)
C15C	0.035 (3)	0.040 (2)	0.039 (2)	−0.005 (2)	−0.001 (2)	−0.007 (2)
C16A	0.052 (3)	0.033 (2)	0.035 (2)	0.010 (2)	−0.008 (2)	−0.0027 (19)
C16B	0.054 (3)	0.046 (3)	0.037 (2)	0.015 (2)	0.008 (2)	0.000 (2)
C16C	0.036 (3)	0.039 (2)	0.044 (3)	−0.005 (2)	−0.007 (2)	0.001 (2)
C17A	0.032 (2)	0.030 (2)	0.033 (2)	0.0022 (18)	0.000 (2)	−0.0002 (18)
C17B	0.037 (3)	0.034 (2)	0.038 (2)	0.002 (2)	0.002 (2)	−0.0025 (19)
C17C	0.031 (3)	0.032 (2)	0.033 (2)	0.0010 (19)	0.0000 (19)	−0.0054 (18)
C18A	0.042 (3)	0.029 (2)	0.037 (2)	−0.004 (2)	0.007 (2)	0.0021 (18)
C18B	0.045 (3)	0.036 (2)	0.044 (3)	0.002 (2)	−0.001 (2)	−0.002 (2)
C18C	0.043 (3)	0.032 (2)	0.036 (2)	0.006 (2)	0.008 (2)	0.0039 (19)
C19A	0.035 (3)	0.030 (2)	0.031 (2)	0.0005 (19)	0.0009 (19)	0.0042 (17)
C19B	0.036 (3)	0.033 (2)	0.040 (2)	−0.006 (2)	0.000 (2)	−0.0066 (19)
C19C	0.039 (3)	0.030 (2)	0.030 (2)	0.0003 (19)	0.000 (2)	0.0009 (18)
C20A	0.034 (3)	0.028 (2)	0.041 (2)	0.0010 (19)	0.006 (2)	−0.0014 (18)
C20B	0.048 (3)	0.032 (2)	0.041 (3)	0.001 (2)	0.001 (2)	−0.003 (2)
C20C	0.036 (3)	0.035 (2)	0.030 (2)	−0.003 (2)	0.002 (2)	−0.0067 (18)
C21A	0.051 (3)	0.027 (2)	0.049 (3)	0.011 (2)	−0.002 (2)	−0.004 (2)
C21B	0.079 (4)	0.038 (3)	0.050 (3)	0.019 (3)	0.009 (3)	0.001 (2)
C21C	0.056 (3)	0.044 (3)	0.054 (3)	−0.015 (3)	−0.012 (3)	0.004 (2)
C22A	0.048 (3)	0.035 (2)	0.037 (2)	0.005 (2)	0.007 (2)	0.0001 (19)
C22B	0.055 (3)	0.038 (2)	0.042 (3)	0.009 (2)	0.002 (2)	−0.003 (2)
C22C	0.055 (3)	0.041 (3)	0.038 (2)	−0.011 (2)	0.005 (2)	−0.008 (2)
C23A	0.040 (3)	0.036 (2)	0.044 (3)	−0.002 (2)	0.010 (2)	−0.004 (2)
C23B	0.071 (4)	0.045 (3)	0.056 (3)	0.013 (3)	−0.010 (3)	−0.004 (3)
C23C	0.060 (3)	0.047 (3)	0.040 (3)	−0.011 (3)	−0.002 (3)	0.003 (2)
C24A	0.036 (3)	0.036 (2)	0.044 (2)	0.004 (2)	0.001 (2)	−0.005 (2)
C24C	0.064 (4)	0.064 (3)	0.045 (3)	−0.020 (3)	−0.001 (3)	0.002 (3)
C25A	0.038 (3)	0.037 (2)	0.055 (3)	0.003 (2)	0.002 (2)	−0.006 (2)
C25C	0.159 (8)	0.061 (4)	0.048 (3)	−0.010 (4)	0.010 (4)	0.012 (3)
C26A	0.077 (4)	0.038 (3)	0.052 (3)	−0.004 (3)	−0.005 (3)	−0.001 (2)
C26C	0.076 (4)	0.047 (3)	0.080 (4)	−0.008 (3)	−0.011 (4)	0.012 (3)
C27A	0.054 (3)	0.045 (3)	0.053 (3)	−0.002 (3)	−0.008 (3)	−0.004 (2)
C27C	0.090 (5)	0.094 (5)	0.052 (3)	−0.004 (4)	0.006 (4)	0.020 (3)
C28A	0.060 (3)	0.033 (3)	0.050 (3)	0.001 (2)	−0.006 (3)	−0.011 (2)
C28B	0.044 (3)	0.028 (2)	0.041 (2)	0.002 (2)	0.005 (2)	−0.007 (2)
C28C	0.034 (3)	0.033 (2)	0.047 (3)	−0.004 (2)	−0.017 (2)	0.003 (2)
C29A	0.059 (3)	0.041 (3)	0.055 (3)	0.008 (2)	−0.010 (3)	−0.013 (2)
C29B	0.081 (4)	0.039 (3)	0.056 (3)	0.000 (3)	−0.018 (3)	0.005 (2)
C29C	0.062 (4)	0.046 (3)	0.056 (3)	−0.019 (3)	−0.003 (3)	0.008 (2)
C30A	0.029 (3)	0.055 (3)	0.049 (3)	0.010 (2)	−0.004 (2)	−0.020 (3)
C30B	0.029 (2)	0.039 (2)	0.039 (2)	0.002 (2)	0.003 (2)	0.001 (2)
C30C	0.051 (3)	0.046 (3)	0.041 (3)	−0.019 (3)	−0.010 (2)	0.018 (2)
C31A	0.033 (3)	0.085 (4)	0.059 (3)	−0.003 (3)	0.007 (3)	−0.019 (3)
C31B	0.032 (3)	0.059 (3)	0.054 (3)	0.001 (2)	−0.006 (2)	−0.005 (3)
C31C	0.044 (3)	0.059 (3)	0.065 (3)	−0.003 (3)	−0.001 (3)	0.019 (3)
O1A	0.0453 (19)	0.0248 (14)	0.0386 (16)	0.0054 (13)	0.0084 (15)	0.0098 (13)
O1B	0.0315 (17)	0.0238 (14)	0.066 (2)	−0.0037 (13)	−0.0086 (16)	−0.0078 (14)
O1C	0.054 (2)	0.0249 (15)	0.067 (2)	−0.0057 (15)	0.0196 (19)	−0.0116 (15)
O2A	0.0394 (18)	0.0319 (15)	0.0370 (16)	0.0021 (14)	0.0106 (15)	0.0021 (13)
O2B	0.0251 (17)	0.0385 (17)	0.072 (2)	−0.0044 (14)	−0.0050 (17)	0.0068 (16)
O2C	0.0397 (19)	0.0372 (17)	0.062 (2)	0.0032 (15)	0.0069 (17)	−0.0024 (16)
O3A	0.052 (2)	0.0349 (16)	0.0454 (19)	0.0099 (15)	−0.0109 (17)	0.0005 (14)
O3B	0.069 (2)	0.0398 (17)	0.0440 (19)	0.0176 (18)	0.0202 (18)	0.0037 (15)
O3C	0.057 (2)	0.0400 (17)	0.0402 (18)	−0.0097 (17)	−0.0074 (17)	0.0001 (14)
O4A	0.0439 (19)	0.0333 (16)	0.0382 (16)	0.0012 (14)	0.0020 (15)	−0.0079 (13)
O4B	0.0453 (19)	0.0274 (15)	0.0492 (18)	−0.0055 (14)	−0.0103 (16)	0.0027 (14)
O4C	0.052 (2)	0.0314 (16)	0.0444 (17)	−0.0047 (15)	0.0062 (17)	0.0057 (14)
O5A	0.165 (5)	0.042 (2)	0.050 (2)	0.010 (3)	−0.002 (3)	0.0033 (19)
O5B	0.070 (3)	0.0261 (17)	0.085 (3)	−0.0068 (17)	−0.022 (2)	−0.0035 (18)
O5C	0.063 (2)	0.0297 (17)	0.069 (2)	−0.0055 (17)	0.004 (2)	0.0033 (18)
O6A	0.0289 (17)	0.0438 (17)	0.0437 (18)	0.0053 (14)	0.0039 (15)	−0.0016 (15)
O6B	0.0241 (16)	0.0368 (16)	0.0449 (17)	0.0014 (13)	0.0037 (14)	−0.0060 (14)
O6C	0.0361 (18)	0.0408 (17)	0.0430 (17)	−0.0118 (15)	−0.0061 (15)	0.0055 (14)
O7A	0.057 (2)	0.058 (2)	0.077 (3)	0.025 (2)	0.003 (2)	−0.014 (2)
O7B	0.0333 (18)	0.0415 (18)	0.066 (2)	0.0061 (15)	0.0030 (17)	−0.0136 (17)
O7C	0.055 (2)	0.048 (2)	0.086 (3)	−0.0241 (19)	0.008 (2)	−0.003 (2)
C24B	0.080 (4)	0.050 (3)	0.044 (3)	0.006 (3)	−0.011 (3)	−0.007 (2)
C25B	0.074 (5)	0.042 (3)	0.051 (4)	0.000 (3)	−0.015 (3)	0.007 (3)
C26B	0.104 (6)	0.058 (4)	0.076 (4)	0.023 (4)	−0.026 (4)	−0.005 (3)
C27B	0.132 (8)	0.060 (4)	0.063 (4)	0.007 (5)	−0.026 (5)	0.014 (4)
C24Z	0.080 (4)	0.050 (3)	0.044 (3)	0.006 (3)	−0.011 (3)	−0.007 (2)
C27Z	0.104 (6)	0.058 (4)	0.076 (4)	0.023 (4)	−0.026 (4)	−0.005 (3)

### Geometric parameters (Å, º)

**Table:**

C1A—C2A	1.527 (6)	C18B—H18E	0.9800
C1A—C10A	1.543 (6)	C18B—H18F	0.9800
C1A—H1A1	0.9900	C18C—H18G	0.9800
C1A—H1A2	0.9900	C18C—H18H	0.9800
C1B—C2B	1.527 (7)	C18C—H18I	0.9800
C1B—C10B	1.544 (6)	C19A—H19G	0.9800
C1B—H1B1	0.9900	C19A—H19H	0.9800
C1B—H1B2	0.9900	C19A—H19I	0.9800
C1C—C2C	1.528 (6)	C19B—H19A	0.9800
C1C—C10C	1.539 (6)	C19B—H19B	0.9800
C1C—H1C1	0.9900	C19B—H19C	0.9800
C1C—H1C2	0.9900	C19C—H19D	0.9800
C2A—C3A	1.514 (6)	C19C—H19E	0.9800
C2A—H2A1	0.9900	C19C—H19F	0.9800
C2A—H2A2	0.9900	C20A—C21A	1.512 (6)
C2B—C3B	1.499 (6)	C20A—C22A	1.540 (6)
C2B—H2B1	0.9900	C20A—H20A	1.0000
C2B—H2B2	0.9900	C20B—C21B	1.510 (7)
C2C—C3C	1.508 (6)	C20B—C22B	1.531 (6)
C2C—H2C1	0.9900	C20B—H20B	1.0000
C2C—H2C2	0.9900	C20C—C21C	1.517 (7)
C3A—O4A	1.460 (5)	C20C—C22C	1.532 (6)
C3A—C4A	1.501 (6)	C20C—H20C	1.0000
C3A—H3A	1.0000	C21A—H21D	0.9800
C3B—O4B	1.460 (5)	C21A—H21E	0.9800
C3B—C4B	1.524 (6)	C21A—H21F	0.9800
C3B—H3B	1.0000	C21B—H21G	0.9800
C3C—O4C	1.474 (5)	C21B—H21H	0.9800
C3C—C4C	1.513 (6)	C21B—H21I	0.9800
C3C—H3C	1.0000	C21C—H21A	0.9800
C4A—C5A	1.539 (6)	C21C—H21B	0.9800
C4A—H4A1	0.9900	C21C—H21C	0.9800
C4A—H4A2	0.9900	C22A—C23A	1.513 (6)
C4B—C5B	1.528 (6)	C22A—H22A	0.9900
C4B—H4B1	0.9900	C22A—H22B	0.9900
C4B—H4B2	0.9900	C22B—C23B	1.527 (6)
C4C—C5C	1.525 (6)	C22B—H22C	0.9900
C4C—H4C1	0.9900	C22B—H22D	0.9900
C4C—H4C2	0.9900	C22C—C23C	1.512 (6)
C5A—O1A	1.441 (5)	C22C—H22E	0.9900
C5A—C10A	1.536 (6)	C22C—H22F	0.9900
C5A—C6A	1.541 (6)	C23A—C24A	1.524 (6)
C5B—O1B	1.432 (5)	C23A—H23A	0.9900
C5B—C6B	1.530 (6)	C23A—H23B	0.9900
C5B—C10B	1.563 (6)	C23B—C24B	1.504 (7)
C5C—O1C	1.435 (5)	C23B—H23C	0.9900
C5C—C10C	1.556 (6)	C23B—H23D	0.9900
C5C—C6C	1.562 (6)	C23C—C24C	1.488 (7)
C6A—O6A	1.462 (5)	C23C—H23E	0.9900
C6A—C7A	1.484 (6)	C23C—H23F	0.9900
C6A—H6A	1.0000	C24A—C25A	1.527 (6)
C6B—O6B	1.469 (5)	C24A—H24A	0.9900
C6B—C7B	1.486 (6)	C24A—H24B	0.9900
C6B—H6B	1.0000	C24C—C25C	1.453 (7)
C6C—O6C	1.415 (6)	C24C—H24C	0.9900
C6C—C7C	1.516 (6)	C24C—H24D	0.9900
C6C—H6C	1.0000	C25A—C27A	1.511 (7)
C7A—C8A	1.344 (6)	C25A—C26A	1.544 (7)
C7A—H7A	0.9500	C25A—H25A	1.0000
C7B—C8B	1.334 (6)	C25C—C26C	1.450 (7)
C7B—H7B	0.9500	C25C—C27C	1.497 (8)
C7C—C8C	1.355 (6)	C25C—H25C	1.0000
C7C—H7C	0.9500	C26A—H26A	0.9800
C8A—C14A	1.494 (5)	C26A—H26B	0.9800
C8A—C9A	1.516 (6)	C26A—H26C	0.9800
C8B—C14B	1.501 (6)	C26C—H26D	0.9800
C8B—C9B	1.535 (6)	C26C—H26E	0.9800
C8C—C14C	1.499 (6)	C26C—H26F	0.9800
C8C—C9C	1.523 (6)	C27A—H27A	0.9800
C9A—O2A	1.449 (5)	C27A—H27B	0.9800
C9A—C11A	1.546 (6)	C27A—H27C	0.9800
C9A—C10A	1.572 (6)	C27C—H27G	0.9800
C9B—O2B	1.443 (5)	C27C—H27H	0.9800
C9B—C11B	1.548 (6)	C27C—H27I	0.9800
C9B—C10B	1.558 (6)	C28A—O5A	1.174 (6)
C9C—O2C	1.434 (5)	C28A—O4A	1.340 (6)
C9C—C11C	1.552 (6)	C28A—C29A	1.483 (7)
C9C—C10C	1.556 (6)	C28B—O5B	1.203 (5)
C10A—C19A	1.543 (6)	C28B—O4B	1.315 (5)
C10B—C19B	1.547 (6)	C28B—C29B	1.471 (7)
C10C—C19C	1.530 (6)	C28C—O5C	1.183 (6)
C11A—O3A	1.210 (5)	C28C—O4C	1.329 (5)
C11A—C12A	1.508 (6)	C28C—C29C	1.500 (7)
C11B—O3B	1.217 (5)	C29A—H29A	0.9800
C11B—C12B	1.506 (6)	C29A—H29B	0.9800
C11C—O3C	1.193 (5)	C29A—H29C	0.9800
C11C—C12C	1.497 (6)	C29B—H29D	0.9800
C12A—C13A	1.536 (6)	C29B—H29E	0.9800
C12A—H12A	0.9900	C29B—H29F	0.9800
C12A—H12B	0.9900	C29C—H29G	0.9800
C12B—C13B	1.541 (6)	C29C—H29H	0.9800
C12B—H12C	0.9900	C29C—H29I	0.9800
C12B—H12D	0.9900	C30A—O7A	1.212 (6)
C12C—C13C	1.530 (6)	C30A—O6A	1.343 (5)
C12C—H12E	0.9900	C30A—C31A	1.472 (7)
C12C—H12F	0.9900	C30B—O7B	1.199 (5)
C13A—C18A	1.531 (6)	C30B—O6B	1.355 (5)
C13A—C14A	1.540 (5)	C30B—C31B	1.489 (7)
C13A—C17A	1.545 (6)	C30C—O7C	1.201 (6)
C13B—C18B	1.518 (6)	C30C—O6C	1.345 (5)
C13B—C14B	1.545 (6)	C30C—C31C	1.505 (7)
C13B—C17B	1.552 (6)	C31A—H31G	0.9800
C13C—C18C	1.526 (6)	C31A—H31H	0.9800
C13C—C14C	1.535 (6)	C31A—H31I	0.9800
C13C—C17C	1.546 (6)	C31B—H31A	0.9800
C14A—C15A	1.515 (6)	C31B—H31B	0.9800
C14A—H14A	1.0000	C31B—H31C	0.9800
C14B—C15B	1.532 (6)	C31C—H31D	0.9800
C14B—H14B	1.0000	C31C—H31E	0.9800
C14C—C15C	1.518 (6)	C31C—H31F	0.9800
C14C—H14C	1.0000	O1A—H1AO	0.9542
C15A—C16A	1.538 (6)	O1B—H1BO	0.8049
C15A—H15A	0.9900	O1C—H1CO	0.8929
C15A—H15B	0.9900	O2A—H2AO	0.9280
C15B—C16B	1.550 (6)	O2B—H2BO	0.8964
C15B—H15E	0.9900	O2C—H2CO	1.0507
C15B—H15F	0.9900	C24B—C25B	1.528 (7)
C15C—C16C	1.528 (6)	C24B—H24E	0.9900
C15C—H15C	0.9900	C24B—H24F	0.9900
C15C—H15D	0.9900	C25B—C26B	1.485 (8)
C16A—C17A	1.561 (6)	C25B—C27B	1.495 (8)
C16A—H16A	0.9900	C25B—H25B	1.0000
C16A—H16B	0.9900	C26B—H26G	0.9800
C16B—C17B	1.545 (6)	C26B—H26H	0.9800
C16B—H16C	0.9900	C26B—H26I	0.9800
C16B—H16D	0.9900	C27B—H27D	0.9800
C16C—C17C	1.565 (6)	C27B—H27E	0.9800
C16C—H16E	0.9900	C27B—H27F	0.9800
C16C—H16F	0.9900	C25Z—C26Z	1.488 (13)
C17A—C20A	1.539 (5)	C25Z—H25Z	1.0000
C17A—H17A	1.0000	C26Z—H26J	0.9800
C17B—C20B	1.542 (6)	C26Z—H26K	0.9800
C17B—H17B	1.0000	C26Z—H26L	0.9800
C17C—C20C	1.531 (6)	O1—C4	1.38 (5)
C17C—H17C	1.0000	O2—C4	1.25 (4)
C18A—H18A	0.9800	C3—C4	1.47 (5)
C18A—H18B	0.9800	C3—H3D	0.9800
C18A—H18C	0.9800	C3—H3E	0.9800
C18B—H18D	0.9800	C3—H3F	0.9800
			
C2A—C1A—C10A	112.4 (3)	C13A—C17A—H17A	107.2
C2A—C1A—H1A1	109.1	C16A—C17A—H17A	107.2
C10A—C1A—H1A1	109.1	C20B—C17B—C16B	113.0 (4)
C2A—C1A—H1A2	109.1	C20B—C17B—C13B	119.1 (4)
C10A—C1A—H1A2	109.1	C16B—C17B—C13B	103.4 (3)
H1A1—C1A—H1A2	107.9	C20B—C17B—H17B	106.9
C2B—C1B—C10B	112.2 (4)	C16B—C17B—H17B	106.9
C2B—C1B—H1B1	109.2	C13B—C17B—H17B	106.9
C10B—C1B—H1B1	109.2	C20C—C17C—C13C	119.6 (3)
C2B—C1B—H1B2	109.2	C20C—C17C—C16C	113.4 (4)
C10B—C1B—H1B2	109.2	C13C—C17C—C16C	102.8 (3)
H1B1—C1B—H1B2	107.9	C20C—C17C—H17C	106.8
C2C—C1C—C10C	113.4 (4)	C13C—C17C—H17C	106.8
C2C—C1C—H1C1	108.9	C16C—C17C—H17C	106.8
C10C—C1C—H1C1	108.9	C13A—C18A—H18A	109.5
C2C—C1C—H1C2	108.9	C13A—C18A—H18B	109.5
C10C—C1C—H1C2	108.9	H18A—C18A—H18B	109.5
H1C1—C1C—H1C2	107.7	C13A—C18A—H18C	109.5
C3A—C2A—C1A	110.7 (3)	H18A—C18A—H18C	109.5
C3A—C2A—H2A1	109.5	H18B—C18A—H18C	109.5
C1A—C2A—H2A1	109.5	C13B—C18B—H18D	109.5
C3A—C2A—H2A2	109.5	C13B—C18B—H18E	109.5
C1A—C2A—H2A2	109.5	H18D—C18B—H18E	109.5
H2A1—C2A—H2A2	108.1	C13B—C18B—H18F	109.5
C3B—C2B—C1B	110.7 (4)	H18D—C18B—H18F	109.5
C3B—C2B—H2B1	109.5	H18E—C18B—H18F	109.5
C1B—C2B—H2B1	109.5	C13C—C18C—H18G	109.5
C3B—C2B—H2B2	109.5	C13C—C18C—H18H	109.5
C1B—C2B—H2B2	109.5	H18G—C18C—H18H	109.5
H2B1—C2B—H2B2	108.1	C13C—C18C—H18I	109.5
C3C—C2C—C1C	109.4 (4)	H18G—C18C—H18I	109.5
C3C—C2C—H2C1	109.8	H18H—C18C—H18I	109.5
C1C—C2C—H2C1	109.8	C10A—C19A—H19G	109.5
C3C—C2C—H2C2	109.8	C10A—C19A—H19H	109.5
C1C—C2C—H2C2	109.8	H19G—C19A—H19H	109.5
H2C1—C2C—H2C2	108.2	C10A—C19A—H19I	109.5
O4A—C3A—C4A	108.3 (3)	H19G—C19A—H19I	109.5
O4A—C3A—C2A	107.8 (3)	H19H—C19A—H19I	109.5
C4A—C3A—C2A	111.0 (3)	C10B—C19B—H19A	109.5
O4A—C3A—H3A	109.9	C10B—C19B—H19B	109.5
C4A—C3A—H3A	109.9	H19A—C19B—H19B	109.5
C2A—C3A—H3A	109.9	C10B—C19B—H19C	109.5
O4B—C3B—C2B	108.0 (4)	H19A—C19B—H19C	109.5
O4B—C3B—C4B	107.6 (3)	H19B—C19B—H19C	109.5
C2B—C3B—C4B	112.0 (4)	C10C—C19C—H19D	109.5
O4B—C3B—H3B	109.7	C10C—C19C—H19E	109.5
C2B—C3B—H3B	109.7	H19D—C19C—H19E	109.5
C4B—C3B—H3B	109.7	C10C—C19C—H19F	109.5
O4C—C3C—C2C	107.7 (4)	H19D—C19C—H19F	109.5
O4C—C3C—C4C	107.0 (3)	H19E—C19C—H19F	109.5
C2C—C3C—C4C	112.1 (4)	C21A—C20A—C17A	113.3 (4)
O4C—C3C—H3C	110.0	C21A—C20A—C22A	109.9 (4)
C2C—C3C—H3C	110.0	C17A—C20A—C22A	110.7 (3)
C4C—C3C—H3C	110.0	C21A—C20A—H20A	107.6
C3A—C4A—C5A	111.7 (3)	C17A—C20A—H20A	107.6
C3A—C4A—H4A1	109.3	C22A—C20A—H20A	107.6
C5A—C4A—H4A1	109.3	C21B—C20B—C22B	110.1 (4)
C3A—C4A—H4A2	109.3	C21B—C20B—C17B	113.5 (4)
C5A—C4A—H4A2	109.3	C22B—C20B—C17B	109.8 (4)
H4A1—C4A—H4A2	107.9	C21B—C20B—H20B	107.7
C3B—C4B—C5B	111.9 (4)	C22B—C20B—H20B	107.7
C3B—C4B—H4B1	109.2	C17B—C20B—H20B	107.7
C5B—C4B—H4B1	109.2	C21C—C20C—C17C	112.2 (4)
C3B—C4B—H4B2	109.2	C21C—C20C—C22C	111.0 (4)
C5B—C4B—H4B2	109.2	C17C—C20C—C22C	110.4 (3)
H4B1—C4B—H4B2	107.9	C21C—C20C—H20C	107.7
C3C—C4C—C5C	112.0 (4)	C17C—C20C—H20C	107.7
C3C—C4C—H4C1	109.2	C22C—C20C—H20C	107.7
C5C—C4C—H4C1	109.2	C20A—C21A—H21D	109.5
C3C—C4C—H4C2	109.2	C20A—C21A—H21E	109.5
C5C—C4C—H4C2	109.2	H21D—C21A—H21E	109.5
H4C1—C4C—H4C2	107.9	C20A—C21A—H21F	109.5
O1A—C5A—C10A	106.1 (3)	H21D—C21A—H21F	109.5
O1A—C5A—C4A	108.8 (3)	H21E—C21A—H21F	109.5
C10A—C5A—C4A	112.7 (3)	C20B—C21B—H21G	109.5
O1A—C5A—C6A	105.0 (3)	C20B—C21B—H21H	109.5
C10A—C5A—C6A	113.7 (3)	H21G—C21B—H21H	109.5
C4A—C5A—C6A	110.0 (3)	C20B—C21B—H21I	109.5
O1B—C5B—C4B	109.2 (3)	H21G—C21B—H21I	109.5
O1B—C5B—C6B	105.7 (3)	H21H—C21B—H21I	109.5
C4B—C5B—C6B	112.0 (3)	C20C—C21C—H21A	109.5
O1B—C5B—C10B	106.2 (3)	C20C—C21C—H21B	109.5
C4B—C5B—C10B	111.5 (3)	H21A—C21C—H21B	109.5
C6B—C5B—C10B	111.8 (3)	C20C—C21C—H21C	109.5
O1C—C5C—C4C	109.0 (3)	H21A—C21C—H21C	109.5
O1C—C5C—C10C	105.7 (3)	H21B—C21C—H21C	109.5
C4C—C5C—C10C	112.6 (3)	C23A—C22A—C20A	112.8 (4)
O1C—C5C—C6C	106.4 (3)	C23A—C22A—H22A	109.0
C4C—C5C—C6C	110.0 (4)	C20A—C22A—H22A	109.0
C10C—C5C—C6C	112.8 (3)	C23A—C22A—H22B	109.0
O6A—C6A—C7A	106.8 (3)	C20A—C22A—H22B	109.0
O6A—C6A—C5A	113.0 (3)	H22A—C22A—H22B	107.8
C7A—C6A—C5A	112.0 (4)	C23B—C22B—C20B	116.0 (4)
O6A—C6A—H6A	108.3	C23B—C22B—H22C	108.3
C7A—C6A—H6A	108.3	C20B—C22B—H22C	108.3
C5A—C6A—H6A	108.3	C23B—C22B—H22D	108.3
O6B—C6B—C7B	107.6 (3)	C20B—C22B—H22D	108.3
O6B—C6B—C5B	111.6 (3)	H22C—C22B—H22D	107.4
C7B—C6B—C5B	114.6 (3)	C23C—C22C—C20C	116.5 (4)
O6B—C6B—H6B	107.6	C23C—C22C—H22E	108.2
C7B—C6B—H6B	107.6	C20C—C22C—H22E	108.2
C5B—C6B—H6B	107.6	C23C—C22C—H22F	108.2
O6C—C6C—C7C	108.0 (4)	C20C—C22C—H22F	108.2
O6C—C6C—C5C	113.2 (4)	H22E—C22C—H22F	107.3
C7C—C6C—C5C	112.2 (4)	C22A—C23A—C24A	115.2 (4)
O6C—C6C—H6C	107.7	C22A—C23A—H23A	108.5
C7C—C6C—H6C	107.7	C24A—C23A—H23A	108.5
C5C—C6C—H6C	107.7	C22A—C23A—H23B	108.5
C8A—C7A—C6A	124.3 (4)	C24A—C23A—H23B	108.5
C8A—C7A—H7A	117.9	H23A—C23A—H23B	107.5
C6A—C7A—H7A	117.9	C24B—C23B—C22B	114.4 (4)
C8B—C7B—C6B	124.8 (4)	C24B—C23B—H23C	108.7
C8B—C7B—H7B	117.6	C22B—C23B—H23C	108.7
C6B—C7B—H7B	117.6	C24B—C23B—H23D	108.7
C8C—C7C—C6C	123.9 (4)	C22B—C23B—H23D	108.7
C8C—C7C—H7C	118.0	H23C—C23B—H23D	107.6
C6C—C7C—H7C	118.0	C24C—C23C—C22C	115.4 (4)
C7A—C8A—C14A	121.7 (4)	C24C—C23C—H23E	108.4
C7A—C8A—C9A	123.1 (4)	C22C—C23C—H23E	108.4
C14A—C8A—C9A	115.2 (4)	C24C—C23C—H23F	108.4
C7B—C8B—C14B	123.6 (4)	C22C—C23C—H23F	108.4
C7B—C8B—C9B	121.3 (4)	H23E—C23C—H23F	107.5
C14B—C8B—C9B	114.9 (3)	C23A—C24A—C25A	114.1 (4)
C7C—C8C—C14C	119.5 (4)	C23A—C24A—H24A	108.7
C7C—C8C—C9C	122.8 (4)	C25A—C24A—H24A	108.7
C14C—C8C—C9C	117.5 (4)	C23A—C24A—H24B	108.7
O2A—C9A—C8A	107.4 (3)	C25A—C24A—H24B	108.7
O2A—C9A—C11A	99.2 (3)	H24A—C24A—H24B	107.6
C8A—C9A—C11A	110.8 (3)	C25C—C24C—C23C	122.9 (5)
O2A—C9A—C10A	112.2 (3)	C25C—C24C—H24C	106.6
C8A—C9A—C10A	113.0 (3)	C23C—C24C—H24C	106.6
C11A—C9A—C10A	113.2 (3)	C25C—C24C—H24D	106.6
O2B—C9B—C8B	107.4 (3)	C23C—C24C—H24D	106.6
O2B—C9B—C11B	100.2 (3)	H24C—C24C—H24D	106.6
C8B—C9B—C11B	110.4 (3)	C27A—C25A—C24A	111.9 (4)
O2B—C9B—C10B	112.2 (3)	C27A—C25A—C26A	110.7 (4)
C8B—C9B—C10B	112.4 (3)	C24A—C25A—C26A	111.2 (4)
C11B—C9B—C10B	113.4 (4)	C27A—C25A—H25A	107.6
O2C—C9C—C8C	106.9 (3)	C24A—C25A—H25A	107.6
O2C—C9C—C11C	98.8 (3)	C26A—C25A—H25A	107.6
C8C—C9C—C11C	109.6 (3)	C26C—C25C—C24C	119.7 (5)
O2C—C9C—C10C	114.7 (3)	C26C—C25C—C27C	116.0 (5)
C8C—C9C—C10C	112.0 (3)	C24C—C25C—C27C	114.0 (6)
C11C—C9C—C10C	113.9 (3)	C26C—C25C—H25C	100.8
C5A—C10A—C1A	108.0 (3)	C24C—C25C—H25C	100.8
C5A—C10A—C19A	110.3 (3)	C27C—C25C—H25C	100.8
C1A—C10A—C19A	110.4 (3)	C25A—C26A—H26A	109.5
C5A—C10A—C9A	108.1 (3)	C25A—C26A—H26B	109.5
C1A—C10A—C9A	111.5 (3)	H26A—C26A—H26B	109.5
C19A—C10A—C9A	108.4 (3)	C25A—C26A—H26C	109.5
C1B—C10B—C19B	110.4 (4)	H26A—C26A—H26C	109.5
C1B—C10B—C9B	111.0 (3)	H26B—C26A—H26C	109.5
C19B—C10B—C9B	109.0 (3)	C25C—C26C—H26D	109.5
C1B—C10B—C5B	108.5 (3)	C25C—C26C—H26E	109.5
C19B—C10B—C5B	109.3 (3)	H26D—C26C—H26E	109.5
C9B—C10B—C5B	108.6 (3)	C25C—C26C—H26F	109.5
C19C—C10C—C1C	110.2 (3)	H26D—C26C—H26F	109.5
C19C—C10C—C9C	109.3 (3)	H26E—C26C—H26F	109.5
C1C—C10C—C9C	111.9 (3)	C25A—C27A—H27A	109.5
C19C—C10C—C5C	109.7 (3)	C25A—C27A—H27B	109.5
C1C—C10C—C5C	107.8 (3)	H27A—C27A—H27B	109.5
C9C—C10C—C5C	107.9 (3)	C25A—C27A—H27C	109.5
O3A—C11A—C12A	119.6 (4)	H27A—C27A—H27C	109.5
O3A—C11A—C9A	122.3 (4)	H27B—C27A—H27C	109.5
C12A—C11A—C9A	118.0 (3)	C25C—C27C—H27G	109.5
O3B—C11B—C12B	120.9 (4)	C25C—C27C—H27H	109.5
O3B—C11B—C9B	123.1 (4)	H27G—C27C—H27H	109.5
C12B—C11B—C9B	116.0 (4)	C25C—C27C—H27I	109.5
O3C—C11C—C12C	121.3 (4)	H27G—C27C—H27I	109.5
O3C—C11C—C9C	122.2 (4)	H27H—C27C—H27I	109.5
C12C—C11C—C9C	116.4 (4)	O5A—C28A—O4A	122.8 (5)
C11A—C12A—C13A	112.8 (3)	O5A—C28A—C29A	125.6 (5)
C11A—C12A—H12A	109.0	O4A—C28A—C29A	111.5 (4)
C13A—C12A—H12A	109.0	O5B—C28B—O4B	122.2 (4)
C11A—C12A—H12B	109.0	O5B—C28B—C29B	125.3 (4)
C13A—C12A—H12B	109.0	O4B—C28B—C29B	112.5 (4)
H12A—C12A—H12B	107.8	O5C—C28C—O4C	124.0 (5)
C11B—C12B—C13B	109.1 (4)	O5C—C28C—C29C	124.5 (4)
C11B—C12B—H12C	109.9	O4C—C28C—C29C	111.6 (4)
C13B—C12B—H12C	109.9	C28A—C29A—H29A	109.5
C11B—C12B—H12D	109.9	C28A—C29A—H29B	109.5
C13B—C12B—H12D	109.9	H29A—C29A—H29B	109.5
H12C—C12B—H12D	108.3	C28A—C29A—H29C	109.5
C11C—C12C—C13C	108.9 (4)	H29A—C29A—H29C	109.5
C11C—C12C—H12E	109.9	H29B—C29A—H29C	109.5
C13C—C12C—H12E	109.9	C28B—C29B—H29D	109.5
C11C—C12C—H12F	109.9	C28B—C29B—H29E	109.5
C13C—C12C—H12F	109.9	H29D—C29B—H29E	109.5
H12E—C12C—H12F	108.3	C28B—C29B—H29F	109.5
C18A—C13A—C12A	109.2 (3)	H29D—C29B—H29F	109.5
C18A—C13A—C14A	111.1 (3)	H29E—C29B—H29F	109.5
C12A—C13A—C14A	106.6 (3)	C28C—C29C—H29G	109.5
C18A—C13A—C17A	112.0 (3)	C28C—C29C—H29H	109.5
C12A—C13A—C17A	116.4 (3)	H29G—C29C—H29H	109.5
C14A—C13A—C17A	101.0 (3)	C28C—C29C—H29I	109.5
C18B—C13B—C12B	109.2 (4)	H29G—C29C—H29I	109.5
C18B—C13B—C14B	112.1 (4)	H29H—C29C—H29I	109.5
C12B—C13B—C14B	106.2 (3)	O7A—C30A—O6A	122.9 (5)
C18B—C13B—C17B	112.2 (4)	O7A—C30A—C31A	125.7 (5)
C12B—C13B—C17B	116.8 (4)	O6A—C30A—C31A	111.4 (5)
C14B—C13B—C17B	99.9 (3)	O7B—C30B—O6B	123.6 (4)
C18C—C13C—C12C	110.2 (4)	O7B—C30B—C31B	125.7 (4)
C18C—C13C—C14C	111.8 (4)	O6B—C30B—C31B	110.6 (4)
C12C—C13C—C14C	107.5 (3)	O7C—C30C—O6C	123.2 (5)
C18C—C13C—C17C	110.1 (3)	O7C—C30C—C31C	125.5 (5)
C12C—C13C—C17C	116.9 (3)	O6C—C30C—C31C	111.2 (4)
C14C—C13C—C17C	99.8 (3)	C30A—C31A—H31G	109.5
C8A—C14A—C15A	119.9 (4)	C30A—C31A—H31H	109.5
C8A—C14A—C13A	112.8 (3)	H31G—C31A—H31H	109.5
C15A—C14A—C13A	104.1 (3)	C30A—C31A—H31I	109.5
C8A—C14A—H14A	106.4	H31G—C31A—H31I	109.5
C15A—C14A—H14A	106.4	H31H—C31A—H31I	109.5
C13A—C14A—H14A	106.4	C30B—C31B—H31A	109.5
C8B—C14B—C15B	119.5 (4)	C30B—C31B—H31B	109.5
C8B—C14B—C13B	115.3 (3)	H31A—C31B—H31B	109.5
C15B—C14B—C13B	103.4 (3)	C30B—C31B—H31C	109.5
C8B—C14B—H14B	105.8	H31A—C31B—H31C	109.5
C15B—C14B—H14B	105.8	H31B—C31B—H31C	109.5
C13B—C14B—H14B	105.8	C30C—C31C—H31D	109.5
C8C—C14C—C15C	120.7 (4)	C30C—C31C—H31E	109.5
C8C—C14C—C13C	114.9 (3)	H31D—C31C—H31E	109.5
C15C—C14C—C13C	104.5 (3)	C30C—C31C—H31F	109.5
C8C—C14C—H14C	105.1	H31D—C31C—H31F	109.5
C15C—C14C—H14C	105.1	H31E—C31C—H31F	109.5
C13C—C14C—H14C	105.1	C5A—O1A—H1AO	116.0
C14A—C15A—C16A	104.0 (4)	C5B—O1B—H1BO	113.3
C14A—C15A—H15A	111.0	C5C—O1C—H1CO	112.8
C16A—C15A—H15A	111.0	C9A—O2A—H2AO	100.6
C14A—C15A—H15B	111.0	C9B—O2B—H2BO	99.3
C16A—C15A—H15B	111.0	C9C—O2C—H2CO	109.1
H15A—C15A—H15B	109.0	C28A—O4A—C3A	117.7 (3)
C14B—C15B—C16B	103.4 (4)	C28B—O4B—C3B	120.2 (4)
C14B—C15B—H15E	111.1	C28C—O4C—C3C	118.0 (4)
C16B—C15B—H15E	111.1	C30A—O6A—C6A	117.4 (4)
C14B—C15B—H15F	111.1	C30B—O6B—C6B	116.4 (3)
C16B—C15B—H15F	111.1	C30C—O6C—C6C	117.9 (4)
H15E—C15B—H15F	109.0	C23B—C24B—C25B	117.8 (4)
C14C—C15C—C16C	103.1 (3)	C23B—C24B—H24E	107.9
C14C—C15C—H15C	111.1	C25B—C24B—H24E	107.9
C16C—C15C—H15C	111.1	C23B—C24B—H24F	107.9
C14C—C15C—H15D	111.1	C25B—C24B—H24F	107.9
C16C—C15C—H15D	111.1	H24E—C24B—H24F	107.2
H15C—C15C—H15D	109.1	C26B—C25B—C27B	110.3 (6)
C15A—C16A—C17A	107.0 (4)	C26B—C25B—C24B	110.9 (5)
C15A—C16A—H16A	110.3	C27B—C25B—C24B	110.8 (5)
C17A—C16A—H16A	110.3	C26B—C25B—H25B	108.2
C15A—C16A—H16B	110.3	C27B—C25B—H25B	108.2
C17A—C16A—H16B	110.3	C24B—C25B—H25B	108.2
H16A—C16A—H16B	108.6	C26Z—C25Z—H25Z	108.9
C17B—C16B—C15B	107.0 (4)	C25Z—C26Z—H26J	109.5
C17B—C16B—H16C	110.3	C25Z—C26Z—H26K	109.5
C15B—C16B—H16C	110.3	H26J—C26Z—H26K	109.5
C17B—C16B—H16D	110.3	C25Z—C26Z—H26L	109.5
C15B—C16B—H16D	110.3	H26J—C26Z—H26L	109.5
H16C—C16B—H16D	108.6	H26K—C26Z—H26L	109.5
C15C—C16C—C17C	107.4 (3)	C4—C3—H3D	109.5
C15C—C16C—H16E	110.2	C4—C3—H3E	109.5
C17C—C16C—H16E	110.2	H3D—C3—H3E	109.5
C15C—C16C—H16F	110.2	C4—C3—H3F	109.5
C17C—C16C—H16F	110.2	H3D—C3—H3F	109.5
H16E—C16C—H16F	108.5	H3E—C3—H3F	109.5
C20A—C17A—C13A	119.1 (3)	O2—C4—O1	119 (4)
C20A—C17A—C16A	112.0 (4)	O2—C4—C3	118 (4)
C13A—C17A—C16A	103.5 (3)	O1—C4—C3	123 (3)
C20A—C17A—H17A	107.2		
			
C10A—C1A—C2A—C3A	−58.2 (5)	C10B—C9B—C11B—O3B	−6.8 (6)
C10B—C1B—C2B—C3B	−57.9 (5)	O2B—C9B—C11B—C12B	−66.5 (4)
C10C—C1C—C2C—C3C	−58.5 (5)	C8B—C9B—C11B—C12B	46.6 (5)
C1A—C2A—C3A—O4A	175.1 (3)	C10B—C9B—C11B—C12B	173.8 (4)
C1A—C2A—C3A—C4A	56.7 (5)	O2C—C9C—C11C—O3C	114.6 (5)
C1B—C2B—C3B—O4B	174.1 (4)	C8C—C9C—C11C—O3C	−133.9 (4)
C1B—C2B—C3B—C4B	55.8 (5)	C10C—C9C—C11C—O3C	−7.5 (6)
C1C—C2C—C3C—O4C	173.7 (3)	O2C—C9C—C11C—C12C	−66.1 (4)
C1C—C2C—C3C—C4C	56.2 (5)	C8C—C9C—C11C—C12C	45.4 (5)
O4A—C3A—C4A—C5A	−173.3 (3)	C10C—C9C—C11C—C12C	171.8 (4)
C2A—C3A—C4A—C5A	−55.2 (5)	O3A—C11A—C12A—C13A	135.9 (4)
O4B—C3B—C4B—C5B	−173.4 (3)	C9A—C11A—C12A—C13A	−47.0 (5)
C2B—C3B—C4B—C5B	−54.9 (5)	O3B—C11B—C12B—C13B	121.3 (5)
O4C—C3C—C4C—C5C	−172.9 (3)	C9B—C11B—C12B—C13B	−59.4 (5)
C2C—C3C—C4C—C5C	−55.1 (5)	O3C—C11C—C12C—C13C	119.5 (5)
C3A—C4A—C5A—O1A	−62.3 (4)	C9C—C11C—C12C—C13C	−59.7 (5)
C3A—C4A—C5A—C10A	55.2 (5)	C11A—C12A—C13A—C18A	−65.1 (4)
C3A—C4A—C5A—C6A	−176.8 (3)	C11A—C12A—C13A—C14A	55.0 (4)
C3B—C4B—C5B—O1B	−62.7 (4)	C11A—C12A—C13A—C17A	166.8 (3)
C3B—C4B—C5B—C6B	−179.5 (3)	C11B—C12B—C13B—C18B	−60.1 (5)
C3B—C4B—C5B—C10B	54.4 (5)	C11B—C12B—C13B—C14B	60.9 (4)
C3C—C4C—C5C—O1C	−63.2 (4)	C11B—C12B—C13B—C17B	171.3 (4)
C3C—C4C—C5C—C10C	53.8 (5)	C11C—C12C—C13C—C18C	−61.2 (4)
C3C—C4C—C5C—C6C	−179.5 (3)	C11C—C12C—C13C—C14C	60.9 (4)
O1A—C5A—C6A—O6A	−166.5 (3)	C11C—C12C—C13C—C17C	172.1 (3)
C10A—C5A—C6A—O6A	78.0 (4)	C7A—C8A—C14A—C15A	−3.9 (6)
C4A—C5A—C6A—O6A	−49.6 (4)	C9A—C8A—C14A—C15A	179.8 (3)
O1A—C5A—C6A—C7A	72.8 (4)	C7A—C8A—C14A—C13A	−127.1 (4)
C10A—C5A—C6A—C7A	−42.8 (5)	C9A—C8A—C14A—C13A	56.6 (5)
C4A—C5A—C6A—C7A	−170.3 (3)	C18A—C13A—C14A—C8A	58.2 (5)
O1B—C5B—C6B—O6B	−158.8 (3)	C12A—C13A—C14A—C8A	−60.6 (5)
C4B—C5B—C6B—O6B	−39.9 (4)	C17A—C13A—C14A—C8A	177.3 (4)
C10B—C5B—C6B—O6B	86.1 (4)	C18A—C13A—C14A—C15A	−73.3 (4)
O1B—C5B—C6B—C7B	78.5 (4)	C12A—C13A—C14A—C15A	167.8 (3)
C4B—C5B—C6B—C7B	−162.6 (3)	C17A—C13A—C14A—C15A	45.7 (4)
C10B—C5B—C6B—C7B	−36.6 (5)	C7B—C8B—C14B—C15B	−13.4 (6)
O1C—C5C—C6C—O6C	−160.8 (3)	C9B—C8B—C14B—C15B	173.1 (4)
C4C—C5C—C6C—O6C	−42.9 (5)	C7B—C8B—C14B—C13B	−137.6 (4)
C10C—C5C—C6C—O6C	83.7 (4)	C9B—C8B—C14B—C13B	48.9 (5)
O1C—C5C—C6C—C7C	76.5 (4)	C18B—C13B—C14B—C8B	61.1 (5)
C4C—C5C—C6C—C7C	−165.5 (4)	C12B—C13B—C14B—C8B	−58.1 (5)
C10C—C5C—C6C—C7C	−38.9 (5)	C17B—C13B—C14B—C8B	−179.9 (4)
O6A—C6A—C7A—C8A	−114.5 (4)	C18B—C13B—C14B—C15B	−71.2 (4)
C5A—C6A—C7A—C8A	9.8 (6)	C12B—C13B—C14B—C15B	169.6 (4)
O6B—C6B—C7B—C8B	−120.5 (4)	C17B—C13B—C14B—C15B	47.8 (4)
C5B—C6B—C7B—C8B	4.3 (6)	C7C—C8C—C14C—C15C	−14.2 (6)
O6C—C6C—C7C—C8C	−118.2 (5)	C9C—C8C—C14C—C15C	171.5 (4)
C5C—C6C—C7C—C8C	7.3 (6)	C7C—C8C—C14C—C13C	−140.8 (4)
C6A—C7A—C8A—C14A	−171.5 (4)	C9C—C8C—C14C—C13C	44.8 (5)
C6A—C7A—C8A—C9A	4.6 (6)	C18C—C13C—C14C—C8C	66.0 (5)
C6B—C7B—C8B—C14B	−168.2 (4)	C12C—C13C—C14C—C8C	−55.1 (5)
C6B—C7B—C8B—C9B	5.0 (6)	C17C—C13C—C14C—C8C	−177.6 (4)
C6C—C7C—C8C—C14C	−172.9 (4)	C18C—C13C—C14C—C15C	−68.6 (4)
C6C—C7C—C8C—C9C	1.2 (7)	C12C—C13C—C14C—C15C	170.3 (3)
C7A—C8A—C9A—O2A	−111.0 (4)	C17C—C13C—C14C—C15C	47.8 (4)
C14A—C8A—C9A—O2A	65.3 (4)	C8A—C14A—C15A—C16A	−162.8 (4)
C7A—C8A—C9A—C11A	141.7 (4)	C13A—C14A—C15A—C16A	−35.5 (4)
C14A—C8A—C9A—C11A	−42.0 (4)	C8B—C14B—C15B—C16B	−165.4 (4)
C7A—C8A—C9A—C10A	13.3 (5)	C13B—C14B—C15B—C16B	−35.6 (5)
C14A—C8A—C9A—C10A	−170.4 (3)	C8C—C14C—C15C—C16C	−167.6 (4)
C7B—C8B—C9B—O2B	−105.0 (4)	C13C—C14C—C15C—C16C	−36.3 (4)
C14B—C8B—C9B—O2B	68.7 (4)	C14A—C15A—C16A—C17A	11.7 (5)
C7B—C8B—C9B—C11B	146.7 (4)	C14B—C15B—C16B—C17B	9.5 (5)
C14B—C8B—C9B—C11B	−39.6 (5)	C14C—C15C—C16C—C17C	10.6 (5)
C7B—C8B—C9B—C10B	18.9 (5)	C18A—C13A—C17A—C20A	−43.8 (5)
C14B—C8B—C9B—C10B	−167.4 (3)	C12A—C13A—C17A—C20A	82.9 (5)
C7C—C8C—C9C—O2C	−104.8 (4)	C14A—C13A—C17A—C20A	−162.2 (4)
C14C—C8C—C9C—O2C	69.4 (4)	C18A—C13A—C17A—C16A	81.2 (4)
C7C—C8C—C9C—C11C	149.1 (4)	C12A—C13A—C17A—C16A	−152.1 (4)
C14C—C8C—C9C—C11C	−36.7 (5)	C14A—C13A—C17A—C16A	−37.1 (4)
C7C—C8C—C9C—C10C	21.7 (5)	C15A—C16A—C17A—C20A	145.7 (4)
C14C—C8C—C9C—C10C	−164.2 (3)	C15A—C16A—C17A—C13A	16.2 (5)
O1A—C5A—C10A—C1A	65.2 (4)	C15B—C16B—C17B—C20B	149.9 (4)
C4A—C5A—C10A—C1A	−53.8 (4)	C15B—C16B—C17B—C13B	19.8 (5)
C6A—C5A—C10A—C1A	−179.9 (3)	C18B—C13B—C17B—C20B	−48.2 (5)
O1A—C5A—C10A—C19A	−174.0 (3)	C12B—C13B—C17B—C20B	78.9 (5)
C4A—C5A—C10A—C19A	67.0 (4)	C14B—C13B—C17B—C20B	−167.2 (4)
C6A—C5A—C10A—C19A	−59.1 (4)	C18B—C13B—C17B—C16B	78.1 (4)
O1A—C5A—C10A—C9A	−55.6 (4)	C12B—C13B—C17B—C16B	−154.8 (4)
C4A—C5A—C10A—C9A	−174.6 (3)	C14B—C13B—C17B—C16B	−40.9 (4)
C6A—C5A—C10A—C9A	59.2 (4)	C18C—C13C—C17C—C20C	−48.3 (5)
C2A—C1A—C10A—C5A	55.7 (4)	C12C—C13C—C17C—C20C	78.5 (5)
C2A—C1A—C10A—C19A	−65.0 (4)	C14C—C13C—C17C—C20C	−166.0 (4)
C2A—C1A—C10A—C9A	174.4 (3)	C18C—C13C—C17C—C16C	78.4 (4)
O2A—C9A—C10A—C5A	78.5 (4)	C12C—C13C—C17C—C16C	−154.8 (4)
C8A—C9A—C10A—C5A	−43.2 (4)	C14C—C13C—C17C—C16C	−39.4 (4)
C11A—C9A—C10A—C5A	−170.3 (3)	C15C—C16C—C17C—C20C	149.0 (4)
O2A—C9A—C10A—C1A	−40.2 (4)	C15C—C16C—C17C—C13C	18.4 (4)
C8A—C9A—C10A—C1A	−161.8 (3)	C13A—C17A—C20A—C21A	−53.9 (5)
C11A—C9A—C10A—C1A	71.1 (4)	C16A—C17A—C20A—C21A	−174.8 (4)
O2A—C9A—C10A—C19A	−162.0 (3)	C13A—C17A—C20A—C22A	−177.9 (4)
C8A—C9A—C10A—C19A	76.4 (4)	C16A—C17A—C20A—C22A	61.2 (5)
C11A—C9A—C10A—C19A	−50.7 (4)	C16B—C17B—C20B—C21B	−177.3 (4)
C2B—C1B—C10B—C19B	−63.4 (5)	C13B—C17B—C20B—C21B	−55.6 (6)
C2B—C1B—C10B—C9B	175.7 (4)	C16B—C17B—C20B—C22B	59.0 (5)
C2B—C1B—C10B—C5B	56.4 (5)	C13B—C17B—C20B—C22B	−179.4 (4)
O2B—C9B—C10B—C1B	−47.0 (5)	C13C—C17C—C20C—C21C	−59.4 (5)
C8B—C9B—C10B—C1B	−168.2 (3)	C16C—C17C—C20C—C21C	179.0 (4)
C11B—C9B—C10B—C1B	65.6 (4)	C13C—C17C—C20C—C22C	176.2 (4)
O2B—C9B—C10B—C19B	−168.8 (3)	C16C—C17C—C20C—C22C	54.6 (5)
C8B—C9B—C10B—C19B	70.0 (4)	C21A—C20A—C22A—C23A	75.7 (5)
C11B—C9B—C10B—C19B	−56.2 (4)	C17A—C20A—C22A—C23A	−158.4 (4)
O2B—C9B—C10B—C5B	72.2 (4)	C21B—C20B—C22B—C23B	57.0 (6)
C8B—C9B—C10B—C5B	−49.0 (4)	C17B—C20B—C22B—C23B	−177.3 (4)
C11B—C9B—C10B—C5B	−175.2 (3)	C21C—C20C—C22C—C23C	57.4 (6)
O1B—C5B—C10B—C1B	64.4 (4)	C17C—C20C—C22C—C23C	−177.5 (4)
C4B—C5B—C10B—C1B	−54.5 (5)	C20A—C22A—C23A—C24A	−174.5 (4)
C6B—C5B—C10B—C1B	179.2 (3)	C20B—C22B—C23B—C24B	176.4 (5)
O1B—C5B—C10B—C19B	−175.1 (3)	C20C—C22C—C23C—C24C	168.0 (5)
C4B—C5B—C10B—C19B	66.0 (4)	C22A—C23A—C24A—C25A	−166.4 (4)
C6B—C5B—C10B—C19B	−60.3 (4)	C22C—C23C—C24C—C25C	170.0 (6)
O1B—C5B—C10B—C9B	−56.4 (4)	C23A—C24A—C25A—C27A	179.6 (4)
C4B—C5B—C10B—C9B	−175.2 (3)	C23A—C24A—C25A—C26A	−56.0 (5)
C6B—C5B—C10B—C9B	58.5 (4)	C23C—C24C—C25C—C26C	30.3 (12)
C2C—C1C—C10C—C19C	−63.6 (5)	C23C—C24C—C25C—C27C	174.1 (6)
C2C—C1C—C10C—C9C	174.6 (3)	O5A—C28A—O4A—C3A	−7.6 (8)
C2C—C1C—C10C—C5C	56.1 (5)	C29A—C28A—O4A—C3A	172.0 (4)
O2C—C9C—C10C—C19C	−169.3 (3)	C4A—C3A—O4A—C28A	−80.4 (5)
C8C—C9C—C10C—C19C	68.7 (4)	C2A—C3A—O4A—C28A	159.5 (4)
C11C—C9C—C10C—C19C	−56.4 (5)	O5B—C28B—O4B—C3B	5.0 (7)
O2C—C9C—C10C—C1C	−46.9 (5)	C29B—C28B—O4B—C3B	−176.5 (4)
C8C—C9C—C10C—C1C	−169.0 (3)	C2B—C3B—O4B—C28B	124.3 (4)
C11C—C9C—C10C—C1C	65.9 (4)	C4B—C3B—O4B—C28B	−114.6 (4)
O2C—C9C—C10C—C5C	71.5 (4)	O5C—C28C—O4C—C3C	−7.0 (7)
C8C—C9C—C10C—C5C	−50.6 (4)	C29C—C28C—O4C—C3C	173.2 (4)
C11C—C9C—C10C—C5C	−175.7 (3)	C2C—C3C—O4C—C28C	143.4 (4)
O1C—C5C—C10C—C19C	−174.1 (3)	C4C—C3C—O4C—C28C	−95.9 (4)
C4C—C5C—C10C—C19C	67.0 (5)	O7A—C30A—O6A—C6A	−5.2 (6)
C6C—C5C—C10C—C19C	−58.2 (5)	C31A—C30A—O6A—C6A	174.4 (4)
O1C—C5C—C10C—C1C	65.9 (4)	C7A—C6A—O6A—C30A	−129.6 (4)
C4C—C5C—C10C—C1C	−53.0 (5)	C5A—C6A—O6A—C30A	106.8 (4)
C6C—C5C—C10C—C1C	−178.2 (3)	O7B—C30B—O6B—C6B	4.8 (6)
O1C—C5C—C10C—C9C	−55.1 (4)	C31B—C30B—O6B—C6B	−173.7 (4)
C4C—C5C—C10C—C9C	−174.0 (3)	C7B—C6B—O6B—C30B	−130.8 (4)
C6C—C5C—C10C—C9C	60.8 (4)	C5B—C6B—O6B—C30B	102.6 (4)
O2A—C9A—C11A—O3A	102.4 (5)	O7C—C30C—O6C—C6C	−3.4 (7)
C8A—C9A—C11A—O3A	−144.9 (4)	C31C—C30C—O6C—C6C	177.2 (4)
C10A—C9A—C11A—O3A	−16.6 (6)	C7C—C6C—O6C—C30C	−127.6 (4)
O2A—C9A—C11A—C12A	−74.6 (4)	C5C—C6C—O6C—C30C	107.5 (4)
C8A—C9A—C11A—C12A	38.1 (5)	C22B—C23B—C24B—C25B	−174.9 (6)
C10A—C9A—C11A—C12A	166.3 (4)	C23B—C24B—C25B—C26B	−56.2 (8)
O2B—C9B—C11B—O3B	112.9 (5)	C23B—C24B—C25B—C27B	−179.1 (7)
C8B—C9B—C11B—O3B	−134.0 (5)		

### Hydrogen-bond geometry (Å, º)

**Table:**

*D*—H···*A*	*D*—H	H···*A*	*D*···*A*	*D*—H···*A*
O1*A*—H1*AO*···O3*C*^i^	0.95	1.79	2.745 (4)	177
O1*B*—H1*BO*···O5*C*	0.80	2.02	2.819 (4)	175
O1*C*—H1*CO*···O5*B*	0.89	1.81	2.699 (4)	178
O2*A*—H2*AO*···O1*A*	0.93	1.88	2.691 (4)	145
O2*B*—H2*BO*···O1*B*	0.90	1.80	2.628 (4)	153
O2*C*—H2*CO*···O1*C*	1.05	1.77	2.614 (4)	134

Symmetry code: (i) −*x*+1, *y*+1/2, −*z*+1/2.
